# Common ancestry of heterodimerizing TALE homeobox transcription factors across Metazoa and Archaeplastida

**DOI:** 10.1186/s12915-018-0605-5

**Published:** 2018-11-05

**Authors:** Sunjoo Joo, Ming Hsiu Wang, Gary Lui, Jenny Lee, Andrew Barnas, Eunsoo Kim, Sebastian Sudek, Alexandra Z. Worden, Jae-Hyeok Lee

**Affiliations:** 1 Department of Botany, University of British Columbia, 6270 University Blvd, Vancouver, BC V6T 1Z4 Canada; 2 Division of Invertebrate Zoology and Sackler Institute for Comparative Genomics, American Museum of Natural History, 200 Central Park West, New York, NY 10024 USA; 3 Monterey Bay Aquarium Research Institute, 7700 Sandholdt Rd, Moss Landing, CA 95039 USA

**Keywords:** Archaeplastida evolution, Developmental mechanism, KNOX transcription factor, PBC-homology, TALE-class homeobox, Transcription factor heterodimerization

## Abstract

**Background:**

Complex multicellularity requires elaborate developmental mechanisms, often based on the versatility of heterodimeric transcription factor (TF) interactions. Homeobox TFs in the TALE superclass are deeply embedded in the gene regulatory networks that orchestrate embryogenesis. Knotted-like homeobox (KNOX) TFs, homologous to animal MEIS, have been found to drive the haploid-to-diploid transition in both unicellular green algae and land plants via heterodimerization with other TALE superclass TFs, demonstrating remarkable functional conservation of a developmental TF across lineages that diverged one billion years ago. Here, we sought to delineate whether TALE-TALE heterodimerization is ancestral to eukaryotes.

**Results:**

We analyzed TALE endowment in the algal radiations of Archaeplastida, ancestral to land plants. Homeodomain phylogeny and bioinformatics analysis partitioned TALEs into two broad groups, KNOX and non-KNOX. Each group shares previously defined heterodimerization domains, plant KNOX-homology in the KNOX group and animal PBC-homology in the non-KNOX group, indicating their deep ancestry. Protein-protein interaction experiments showed that the TALEs in the two groups all participated in heterodimerization.

**Conclusions:**

Our study indicates that the TF dyads consisting of KNOX/MEIS and PBC-containing TALEs must have evolved early in eukaryotic evolution. Based on our results, we hypothesize that in early eukaryotes, the TALE heterodimeric configuration provided transcription-on switches via dimerization-dependent subcellular localization, ensuring execution of the haploid-to-diploid transition only when the gamete fusion is correctly executed between appropriate partner gametes. The TALE switch then diversified in the several lineages that engage in a complex multicellular organization.

**Electronic supplementary material:**

The online version of this article (10.1186/s12915-018-0605-5) contains supplementary material, which is available to authorized users.

## Background

The homeobox transcription factors (TFs) are ubiquitous in eukaryotes, carrying a DNA-binding homeodomain typically 60 amino acids that folds into three α-helices [1]. The atypical or TALE (three-amino acid length extension) superclass of homeobox TFs shares a three-amino-acid insertion between helix 1 and 2 and plays essential roles during embryonic development by participating in interactive TF networks. In animals, MEIS- and PBC-class TALE proteins, such as Meis/Hth and Pbx/Exd, form heterodimers that in turn form ternary complexes with HOX-class homeobox TFs, determining cellular fates along the anterior-posterior axis of the developing embryo [2, 3]. In plants, the interacting KNOX- and BELL-class TFs in the TALE group play critical roles during organ formation and the vegetative-to-reproductive transition in the undifferentiated cell mass known as the shoot apical meristem [4, 5].

The heterodimerization of TALE proteins serves as a trigger for precise execution of developmental programs. Prior to heterodimerization, animal PBX proteins are localized in the cytosol, and upon binding to MEIS, they translocate to the nucleus [6, 7]. Similar heterodimerization-dependent translocation is also observed for KNOX-BELL pairs in the plant *Arabidopsis*, implying that this mechanism is a conserved regulatory feature of TALE proteins [8]. In addition, TALE proteins differ in their DNA-binding specificity [9, 10], which is primarily determined by the homeodomain residues at positions 47, 50, and 54 [11], and heterodimerization increases target affinity by bringing two such DNA-binding domains together.

TALE-heterodimerization is mediated by class-specific homology domains located on the N-terminal side adjacent to the homeodomain [12, 13]. Animal MEIS and plant KNOX class proteins share readily identifiable homology in their heterodimerization domain, leading to the proposal of an ancestral TALE class named MEINOX [12]. In contrast, their partner classes—PBC and BELL—exhibit no apparent sequence similarity in their heterodimerization domains. Short shared sequence motifs and common secondary structures have been found within the heterodimerization domains between MEINOX and PBC or BELL [14, 15], but their extent of the homology requires adequate taxon sampling to recover ancestral relationships.

An ancestral function of TALE-TALE heterodimerization was revealed in studies of the unicellular green alga *Chlamydomonas reinhardtii*: the KNOX ortholog GSM1 and a second TALE protein GSP1 form heterodimers immediately after the fusion of sexual gametes, and these drive the haploid-to-diploid transition by activating > 200 diploid-specific genes and inactivating > 100 haploid-specific genes [10, 16, 17]. In subsequent studies, plant-type TALE-TALE heterodimers between KNOX and BELL were shown to be required for the haploid-to-diploid transition of the moss *Physcomitrella patens* [18, 19]. Given the conserved role of TALE heterodimerization as a developmental switch in the sexual life cycle of the plant lineage, understanding its origins and diversification promises to shed light on the evolution of developmental mechanisms during eukaryotic radiation and the emergence of land plants.

**Fig. 1:**
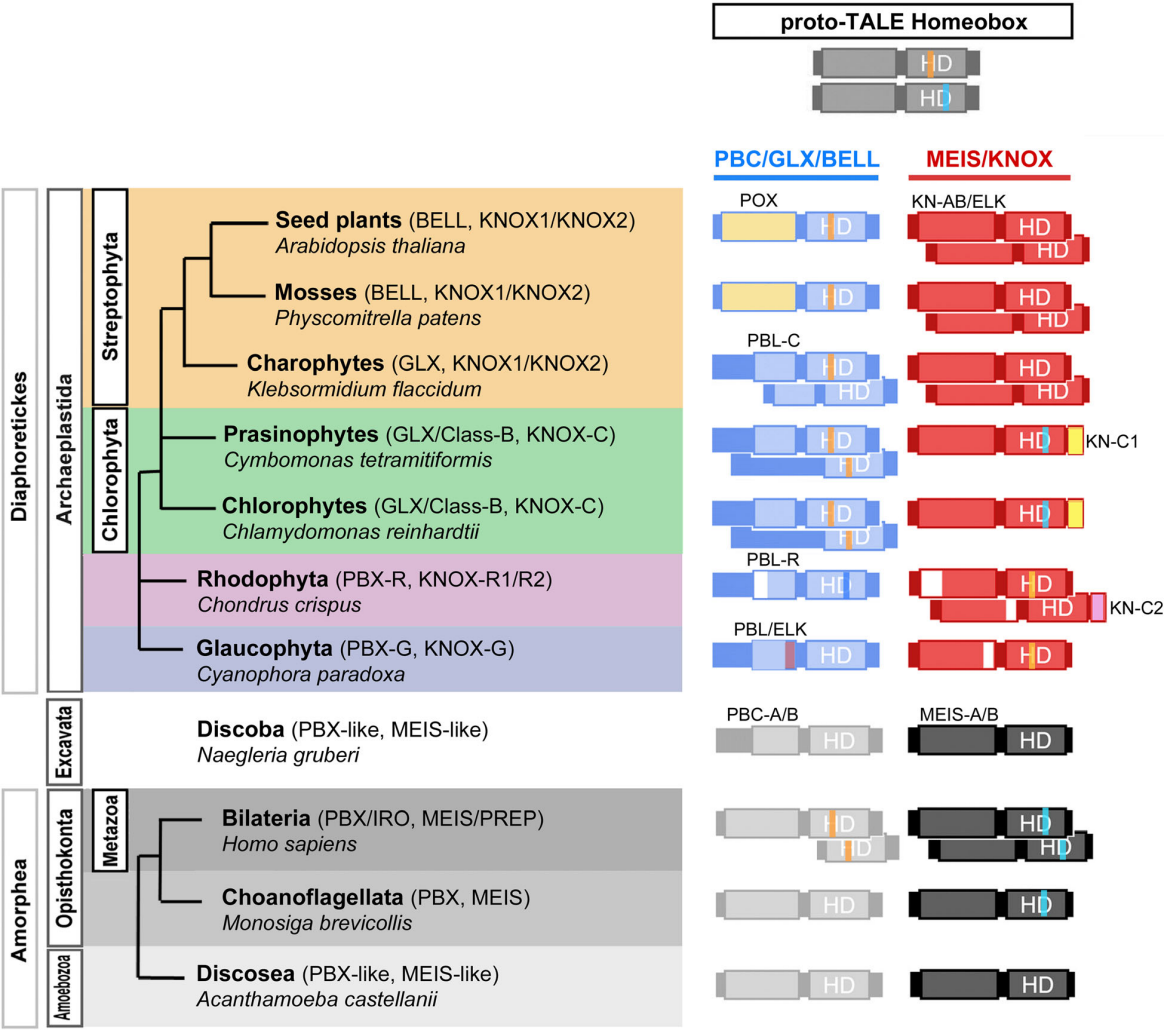
Common origin of heterodimerizing TALE homeobox TFs. Hypothesized homodimerizing proto-TALE protein (top) duplicated before the eukaryotic radiations into animals/fungi/amoebae vs. algae/plants. Lineage-specific diversification soon followed, generating heterodimeric configurations distinct at the phylum-level. (Left) Each lineage possesses one or two classes of potential heterodimeric TALEs, which are summarized onto the eukaryotic phylogeny. A representative species name is given for each analyzed lineage. (Right) Summary of TALE configurations, coupling members of the PBC/PBX/GLX group that shares PBC-homology domains and of the MEIS/KNOX group that shows homology in the KN-A/B domains N-terminal to the homeodomain. Lightly shaded boxes depict homology domains, whose names are provided above. Open areas in the domain boxes indicate the absence of MEINOX-motif for PBX-Red, KN-A for KNOX-Red1 and ELK for KNOX-Red2. Colored vertical lines in the HD indicate two shared introns at 44/45 (orange over “H” in HD) and 48(2/3) (blue over “D” in HD), whose alternating existence between the two groups suggests independent diversification of TALE heterodimerization. HD: Homeodomain; PBL-C: PBL-Chloro; PBL-R: PBL-Red

The Archaeplastida consists of three monophyletic phyla [20, 21] (Fig. 1). (1) Viridiplantae include two divisions, Chlorophyta—chlorophytes and prasinophytes (a paraphyletic group of seven lineages [22])—and Streptophyta—charophyte algae and land plants [23]. (2) Rhodophyta (red algae) include diverse unicellular and multicellular organisms that diverge into four major lineages [24] (Additional file 1: Table S1). (3) Glaucophyta members include only four cultured genera and possess plastids that carry ancestral features of the cyanobacterial symbiont that gave rise to photosynthetic organelles in eukaryotes [25].

To delineate the ancestry of plant-type TALE heterodimerization, we performed a phylogenetic and bioinformatics analysis of TALE TFs in the three algal radiations of the Archaeplastida supergroup, the descendants of a single endosymbiosis event greater than one billion years ago [26, 27]. Our analysis showed that the TALEs were already diversified into two groups at the origin of Archaeplastida, one sharing KNOX-homology and the other sharing PBC-homology. Together with our protein-protein interaction data, we propose that all TALE classes participate in heterodimerization networks via the KNOX- and PBC-homology domains between the two ancestral groups.

## Results

### TALEs in Archaeplastida are divided into two groups, KNOX and non-KNOX

To collect all the available homeobox protein sequences, we performed BLAST and Pfam-motif searches against non-plant genomes and transcriptome assemblies throughout the Archaeplastida, identifying 338 proteins from 56 species as the Archaeplastida homeobox collection (30 genomes and 18 transcriptomes; Additional file 1: Table S1). Of these, 104 possessed the defining feature of TALE proteins, a three-amino-acid insertion between aa positions 23–24 in the homeodomain [28]. At least two TALE genes were detected in most genomes except five genomes in the Trebouxiophyceae class of the Chlorophyta (Additional file 1: Table S1; see Additional file 2: Note S1 for further discussion of the absence of TALEs in Trebouxiophyceae).

**Fig. 2:**
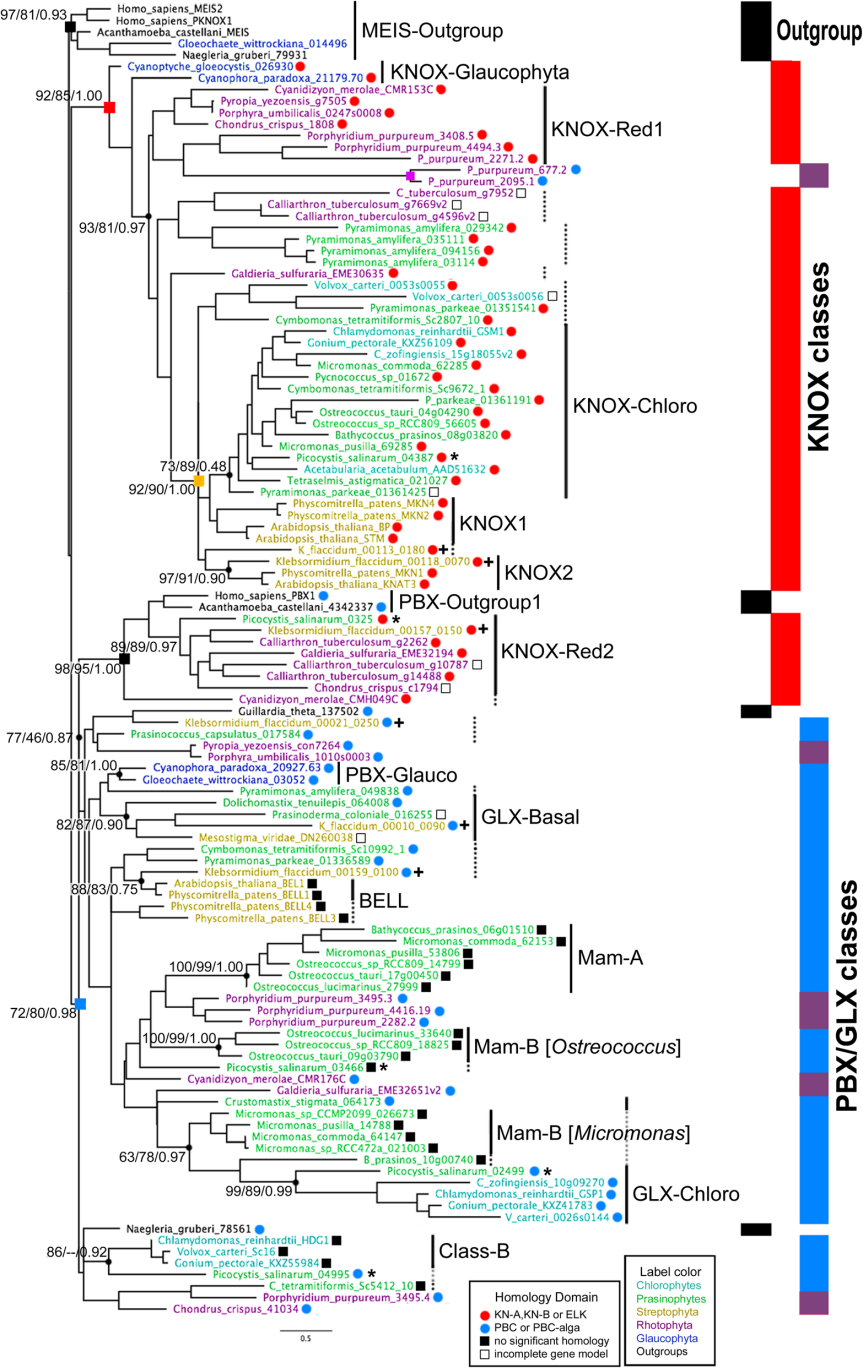
Maximum likelihood (ML) phylogeny of the TALE superclass homeodomain in Archaeplastida supports ancient division between KNOX- and non-KNOX TALE groups. The ML trees were generated from the homeodomain alignment with 70 amino acid positions. The consensus tree out of 1000 bootstrap trees is shown. The three numbers at critical nodes show %bootstrap, %SH, and Bayesian posterior probability in support of clades. The tree contains two outgroup clades marked by black squares at nodes, and two Archaeplastida clades, one combining most KNOX sequences marked by the red square and the other combining all non-KNOX sequences marked by the blue square. Vertical bars on the right depict the distribution of outgroup in black, KNOX in red, and non-KNOX sequences in blue. Red dots by the sequence names indicate the presence of KN-A or KN-B domains, and blue dots indicate the presence of a PBC-homology domain. Truncated sequences not available for homology domain analysis are marked with open black boxes. Filled black boxes indicate the absence of a KN-A/B or PBC-homology domain. Proposed classification is indicated by the vertical lines. Dotted vertical lines indicate suggested class members placed outside the main clade for the class in the phylogeny. PBX-Red sequences are found in four separate clades, marked by purple shades on the blue section of the vertical bars. Two PBX-Red sequences marked by the purple square are exceptionally found in the KNOX-Red1 clade, having divergent amino acids at highly conserved positions at Trp19, His23, and Lys31 in their homeodomain, suggesting their false association with the KNOX-Red1. Colors of the sequence names indicate their phylogenetic group: Blue for Glaucophyta, purple for Rhodophyta, green for prasinophytes, light blue for the chlorophytes, orange for Streptophyta, and black for outgroups. The ruler shows genetic distance. Details of the sequences analyzed by this phylogeny are provided in Additional file 1: Table S2. *Gloeochaete_wittrockiana_014496 is considered as a sequence from a bannelid-type amoeba that contaminated the original culture (SAG46.84) for the MMETSP1089 transcriptome. **Association of KNOX-Red2 class sequences to Amorphea PBC sequences is attributed to a shared WFGN motif determining DNA-binding specificity of the homeodomain via convergent evolution

The collected TALE sequences were then classified by their homeodomain features using a phylogenetic approach, with TALEs from animals, plants, and early-diverging eukaryotes (Amoebozoa and Excavata) as outgroups (Additional file 3: Figure S1). The resultant TALE homeodomain phylogeny distinguished two groups in all three phyla of Archaeplastida (Fig. 2). (1) The KNOX-group as a well-supported clade displayed a phylum-specific cladogram: two Glaucophyta sequences at the base (as KNOX-Glauco) were separate from the next clade, which combines Rhodophyta sequences (as KNOX-Red1) and a Viridiplantae-specific clade with strong support (92/90/1.00). (2) The non-KNOX group, including the BELL and GSP1 homologs, contained clades of mixed taxonomic affiliations. These analyses showed that the TALE proteins had already diverged into two groups before the evolution of the Archaeplastida and that the KNOX-group is highly conserved throughout Archaeplastida.

### KNOX group sequences share the same heterodimerization domains throughout Archaeplastida

The next question was whether the plant KNOX class originated prior to the Viridiplantae phylum. The plant KNOX proteins and the Chlorophyta GSM1 possess the KNOX-homology, consisting of KN-A, KN-B, and ELK domains, required for their heterodimerization with other TALE proteins [10]; therefore, the presence of the KNOX homology would suggest the potential for heterodimerization to the KNOX group. To collect homology domains without prior information, we performed ad-hoc homology domain searches among the KNOX group sequences. Using the identified homology domains as anchors, we carefully curated an alignment of the KNOX-group sequences combined with any other TALE sequences with a KNOX-homology, (Additional file 3: Figure S2). From this KNOX alignment, we found all KNOX group sequences (excluding partial sequences) showing amino acid similarity scores > 50% for at least two of the three domains comprising the KNOX-homology region (Additional file 1: Table S3 for calculated domain homology). To test whether the observed similarity is specific to the TALE sequences, we generated HMM motifs for KN-A and KN-B domains from the KNOX alignment, searched them in the target genomes, and confirmed that KN-A and KN-B domains are found only in the TALE sequences (Additional file 4: Data S1 and S2). We thereby defined KNOX-homologs as the TALE sequences possessing searchable KNOX homology (Fig 2, marked by red dots following their IDs), suggesting that the KNOX-homolog already existed before the evolution of eukaryotic photosynthesis as represented by the Archaeplastida.

In addition to the KNOX-homology, the same search also revealed two novel domains at the C-terminus of the homeodomain (Additional file 3: Figure S2): the first (KN-C1) was shared among the Chlorophyta sequences, and the second (KN-C2) was shared among a group of KNOX homologs in a clade outside the KNOX-group (KNOX-Red2).

### KNOX classes diverged independently among the algal phyla

In Viridiplantae, we found a single KNOX homolog in most Chlorophyta species, whereas KNOX1 and KNOX2 divergence was evident in the Streptophyta division, including the charophyte *Klebsormidium flaccidum* and land plants (Fig. 2). The newly discovered KN-C1 domain was specific to the Chlorophyta KNOX sequences and found in all but one species (*Pyramimonas amylifera*). The absence of similarity between KN-C1 and the C-terminal extensions of KNOX1/KNOX2 sequences suggests independent, lineage-specific KNOX evolution in the Chlorophyta and Streptophyta (Additional file 3: Figure S2). We, therefore, refer to the Chlorophyta KNOX classes as KNOX-Chloro in contrast to the KNOX1 and KNOX2 classes in the Streptophyta.

The KNOX homologs in the Rhodophyta were divided into two classes: a paraphyletic group close to the KNOX-Chloro clade, named KNOX-Red1, and a second group near the PBX-Outgroup, named KNOX-Red2. KNOX-Red1 lacked a KN-A, whereas KNOX-Red2 lacked an ELK and shared a KN-C2 domain (Additional file 3: Figure S2). We consider KNOX-Red1 as the ancestral type, since the KNOX-Red1 sequences were found in all examined Rhodophyta taxa, whereas the KNOX-Red2 sequences were restricted to two taxonomic classes (Cyanidiophyceae and Florideophyceae). Interestingly, the KNOX-Red2 clade included two green algal sequences, with strong statistical support (89/89/0.97; Fig. 2); these possessed a KN-C2 domain, suggesting their ancestry within the KNOX-Red2 class (Additional file 3: Figure S2; see Additional file 2: Note S2 for further discussion about their possible origin via horizontal gene transfer).

Available TALE sequences were limited for the Glaucophyta. We found a single KNOX homolog in two species, which possessed KN-A and KN-B domains but lacked an ELK domain. We termed these KNOX-Glauco.

### Non-KNOX group TALEs possess animal type PBC-homology domain, suggesting a shared ancestry between Archaeplastida and Metazoa

Following the identification of KNOX homologs, the remaining TALE sequences were combined as the non-KNOX group that lacks KN-A and KN-B domains in Archaeplastida. Further classification of the non-KNOX group was challenging due to its highly divergent homeodomain sequences. However, we noticed that the number of non-KNOX genes per species was largely invariable: one in most Rhodophyta and Glaucophyta genomes and two in the majority of Chlorophyta genomes, suggesting their conservation within each radiation.

**Fig. 3:**
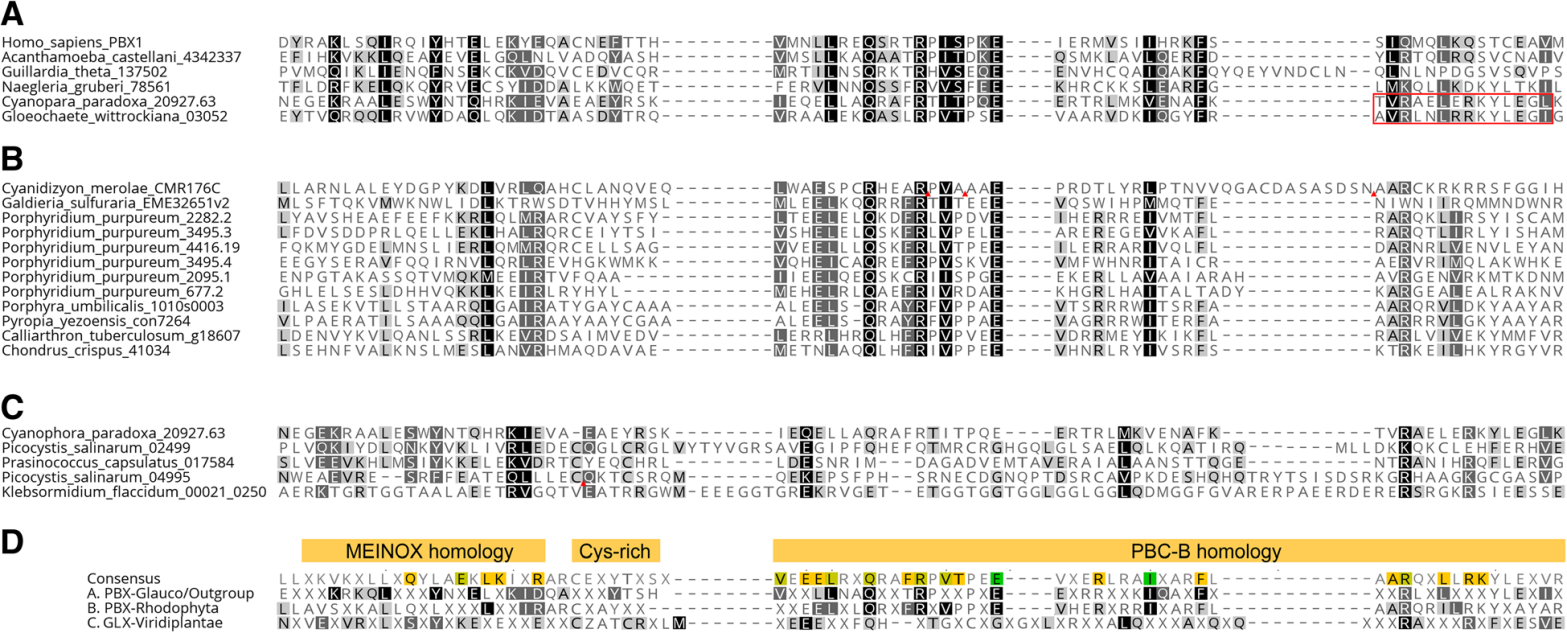
Archaeplastida non-KNOX group TALEs possess a PBC-like domain (PBL) consisting of N-terminal MEINOX homology and C-terminal PBC-B homology. Amino acid letters in black with gray shades, in white with light shades, and in white with black shades show more than 60%, 80%, or 100% similarity in each column. Inverse red triangles indicate the discarded sequences in un-aligned insertions. **a** PBL-Glauco domain alignment, including two Glaucophyta sequences sharing homology in both MEINOX homology and C-terminal half of the PBC-B domain with non-Archaeplastida TALE sequences. Red box indicates the ELK domain. **b** PBL-Red domain alignment. All Rhodophyta non-KNOX sequences possess a PBL domain with poor MEINOX homology. **c** PBL-Chloro domain alignment. Cyanophora_paradox_20927.63 is included for comparison. Picocystis_salinarum_02499 is a founding member of GLX class with a PBL-Chloro domain. **d** Comparison among PBL domains. The top row shows the consensus made from the alignment of (**a**), (**b**), and (**c**) combined and the lower consensus sequences are collected from the individual alignments presented in (**a**), (**b**), and (**c**)

Our ad-hoc homology search provided critical information for non-KNOX classification, identifying a homology domain shared among all Glaucophyta and Rhodophyta non-KNOX sequences (Fig. 3a, b). Since this domain showed a similarity to the second half of the animal PBC-B domain (Pfam ID: PF03792) known as heterodimerization domain [12], we named this domain PBL (PBC-B Like). Accordingly, we classified all the non-KNOX TALEs in Glaucophyta and Rhodophyta as a single PBC-related homeobox class, PBX-Glauco or PBX-Red. PBX-Glauco sequences also possessed the MEINOX motif, conserved in the animal PBC-B domain, indicating common ancestry of PBC-B and PBL domains (Fig. 3a).

### GSP1 shares distant PBC-homology together with other non-KNOX group sequences in Viridiplantae

A remaining question was the evolution of the Chlorophyta non-KNOX sequences that apparently lacked a PBC-homology. To uncover even a distant homology, we compared the newly defined PBL domains with the Chlorophyta sequences by BLAST (cut-off *E*-value of 1E-1) and multiple sequence alignments. This query collected three prasinophyte and one charophyte TALE sequences that possessed a MEINOX motif and a putative PBL-domain; however, they showed very low sequence identity among themselves (Fig. 3c). Further query utilizing these four sequences identified 11 additional non-KNOX sequences. Nine of these were made into two alignments, one including GSP1 homologs and the other combining most prasinophyte sequences (Additional file 3: Figure S3). The two remaining sequences (Picocystis_salinarum_04995 and Klebsormidium_flaccidum_00021_0250) showed a homology to a PBX-Red sequence of *Chondrus cruentum* (ID:41034) in a ~ 200 aa-long extension beyond the PBL domain, suggesting their PBX-Red ancestry (another potential case of horizontal transfers; Additional file 3: Figure S4). All the Chlorophyta non-KNOX sequences that carry the PBL-homology domains were classified as GLX (GSP1-like homeobox) in recognition of the GSP1 protein of *Chlamydomonas* as the first characterized member of this class [29].

### Is the plant BELL class homologous to the Chlorophyta GLX class?

The BELL class is the only non-KNOX class in land plants, sharing a POX (Pre-homeobox) domain (PF07526) [13] and lacking an identifiable PBL domain. The *K. flaccidum* genome, one of the two genomes available in the charophyte from which land plant emerged, contained three non-KNOX sequences, all possessing a PBL domain (Fig. 3, Additional file 3: Figures S3, S4). The second charophyte genome of *Chara braunii* contained one putative BELL homolog that appears to be truncated for the N-terminal sequences outside its C-terminal homeodomain possibly due to the incomplete gene model. Therefore, the lack of PBL-homology in the plant BELL class appears to be due to divergence or domain loss from an old charophyte class that had PBL-homology. We found an intron at the 24(2/3) homeodomain position of a *K. flaccidum* GLX homolog, which was previously identified as being specific to the plant BELL class (Additional file 3: Figure S5) [12], suggesting that the plant BELL class evolved from an ancestral GLX gene. More taxon sampling in charophytes is needed to confirm this inference.

### Two non-KNOX paralogs of Chlorophyta heterodimerize with the KNOX homologs

Even with our sensitive iterative homology search, we could not identify a PBC/PBL-homology in about half of the Chlorophyta non-KNOX sequences. Since most Chlorophyta genomes possess one GLX homolog and one non-KNOX sequence without the PBL-homology domain, we refer the latter collectively to Class-B (Additional file 3: Figure S6). Exceptions were found in one prasinophyte clade (class Mamiellophyceae), whose six high-quality genomes all contain two non-KNOX sequences lacking the PBL-homology. Nonetheless, these non-KNOX sequences formed two groups, one more conserved and the other less conserved and polyphyletic, referred to the Mam-A and Mam-B classes, respectively (Additional file 3: Figures S7, S8). Considering the reductive genome evolution of the Mamiellophyceae [30], the conserved Mam-A class may be derived from an ancestral GLX class.

Two divergent non-KNOX classes in Chlorophyta led to a critical question about their dyadic networks. Previously studies had shown that TALE heterodimers required interaction between MEIS and PBC domains in animals and between KNOX and PBL domains in *Chlamydomonas* [6, 10]. It was, therefore, predicted that all Glaucophyta and Rhodophyta TALEs form heterodimers via their KNOX- and PBL-homology domains. On the other hand, it remained to be tested whether the Chlorophyta TALEs lacking a PBL-domain can form heterodimers with other TALEs.

**Fig. 4:**
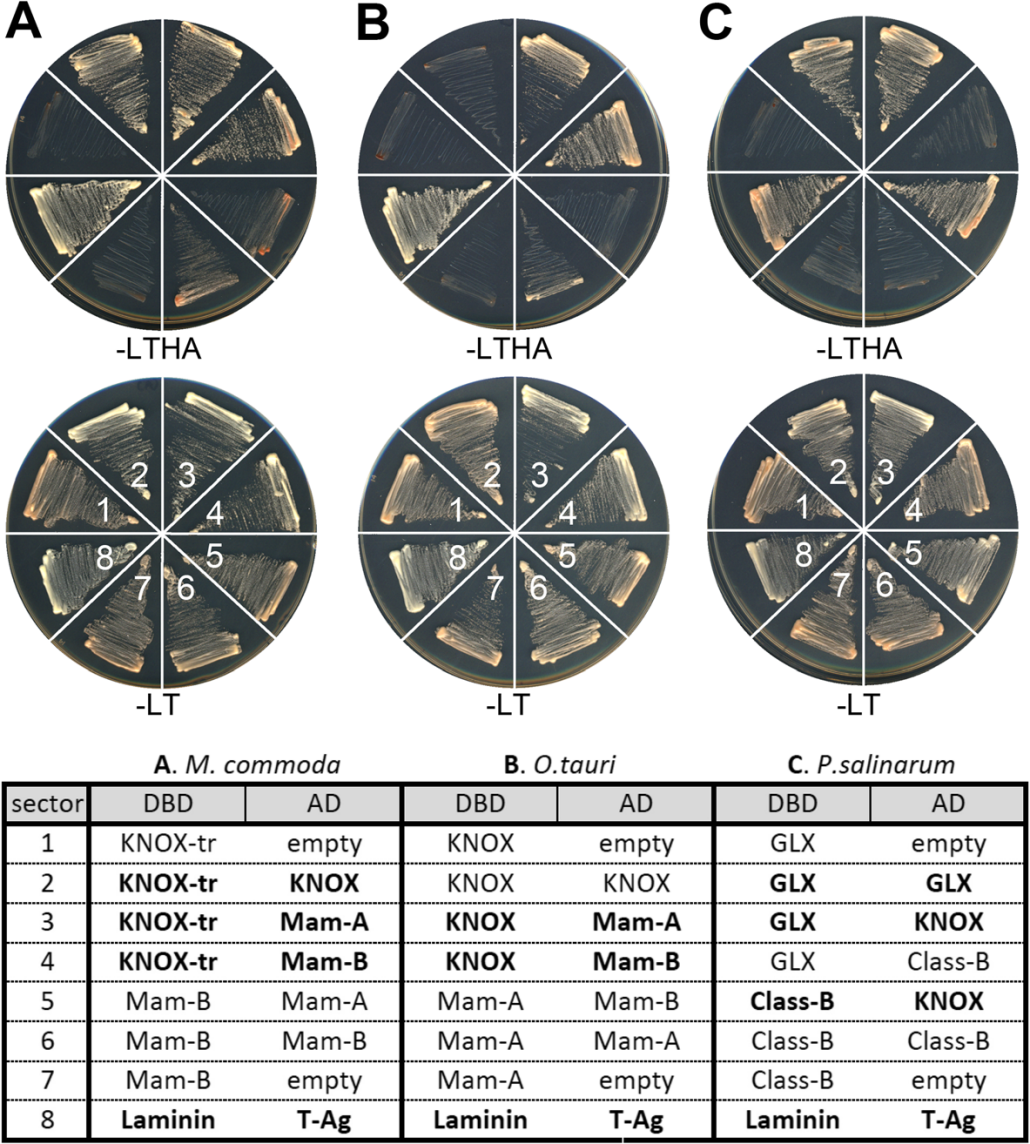
TALE TFs engage in heterodimerization networks between KNOX and non-KNOX groups. The bait constructs conjugated to the GAL4 DNA-binding domain (DBD) and the prey constructs conjugated to the GAL4 transcriptional activation domain (AD) are listed in the table. Construct combinations, numbered 1–8, are arranged in wedges clock-wise, starting at 9 o’clock as labeled in the -LT panels. Confirmed interacting pairs are shown in bold faces in the table. The laminin and T-Antigen (T-Ag) pair, known to be interacting partners, was plated in the 8th sector as a positive control. **a** Assays using *M. commoda* TALEs. **b** Assays using *O. tauri* TALEs. **c** Assays using *P. salinarum* TALEs. KNOX-tr refers to the N-terminal truncated KNOX construct for preventing self-activation. **d** Detailed construct information is provided in Additional file 1: Table S5

To characterize the interaction network of TALE class proteins in Chlorophyta, we selected three prasinophyte species for protein-protein interaction assays: two species containing Mam-A and Mam-B genes (*Micromonas commoda* and *Ostreococcus tauri*), and another species (*Picocystis salinarum*), whose transcriptome contained one GLX and one Class-B sequence. In all three species, we found that KNOX homologs interacted with all examined non-KNOX proteins in Mam-A, Mam-B, Class-B, and GLX class (Fig. 4a–c). No interaction was observed between the two non-KNOX proteins in any of the three species (Fig. 4a–c). Similar to the GLX-KNOX heterodimerization, Mam-A and Mam-B also required additional domains outside the homeodomain for their heterodimerization with the KNOX homologs (Additional file 3: Figure S9). These results showed that the all divergent non-KNOX TALEs maintained their original activity to form heterodimers with the KNOX homologs. Observed interacting network among the TALE sequences is summarized in Additional file 3: Figure S10.

### TALE heterodimerization evolved early in eukaryotic history

Our discovery of the PBC-homology in Archaeplastida suggests common ancestry of the heterodimerizing TALES between Metazoa and Archaeplastida. It also predicted that other eukaryotic lineages might possess TALEs with the PBC-homology. Outside animals, the Pfam database contains only two PBC-B domain-harboring sequences, one from a Cryptophyta species (*Guillardia theta,* ID:137502) and the other from an Amoebozoa species (*Acanthamoeba castillian,* ID:XP_004342337) [31]. We further examined the Excavata group, near to the posited root of eukaryotic phylogeny [20]. A search of two genomes (*Naegleria gruberi* and *Bodo saltans*) collected 12 TALE homeobox sequences in *N.gruberi*, and none in *B.saltans*, of which we found one with a PBC-homology domain (ID:78561, Fig. 3a) and one with a MEIS/KNOX-homology (ID:79931, Additional file 3: Figure S2). We searched additional genomes in the Amorphea and found the PBC-homology and MEIS/KNOX-homology in the TALE sequences collected from Apusozoa, Ichtyhosporea, and Choanoflagellata but not from Fungi (Additional file 3: Figures S11-S14). Our data suggest that the heterodimerization domains—the PBC-homology and MEIS/KNOX-homology—originated early in eukaryotic evolution and persisted throughout the major eukaryotic radiations.

### Intron-retention supports the parallel evolution of the heterodimeric TALE classes during eukaryotic radiations

The ubiquitous presence of dyadic TALEs raised next question: Are all the dyadic TALEs reported in this study the descendants of a single ancestral dyad, or do they result from lineage-specific evolution from a single prototypical TALE (proto-TALE) that does not engage in heterodimerization. To probe deep ancestry, we examined intron-retention, this being regarded as a long-preserved character and less prone to occur by homoplasy (a character displayed by a set of species but not present in their common ancestor) [32]. Five intron positions were shared by at least two TALE classes, of which the 44/45 and 48(2/3) introns qualified as the most ancestral since they were found throughout the Archaeplastida and Metazoa (Additional file 3: Figure S5).

The 44/45 and 48(2/3) introns showed an intriguing exclusive distribution between the two dyadic partners of each phylum: one possesses the 44/45 and the other possesses the 48(2/3) intron (Additional file 3: Figure S5). This mutually exclusive pattern suggested that two TALE genes with distinct intron positions existed at the onset of the eukaryotic radiation. We consider the 44/45 intron position as the most ancestral, given that it was conserved in most non-TALE homeobox genes [12]. In this regard, we speculate that acquisition of the 48(2/3), and loss of the 44/45 intron, accompanied an early event wherein the proto-TALE with the 44/45 intron was duplicated to generate a second TALE with the 48(2/3) intron. Since the 48(2/3) intron position was found within the KNOX/MEIS group genes in Viridiplantae and Metazoa and also in the PBX group genes in Rhodophyta and Cryptophyta, we may speculate that the duplicated TALEs arose early and diversified to establish lineage-specific heterodimeric configurations during eukaryotic radiations. Alternatively, the 48(2/3) intron position in the TALE homeodomain might have been acquired many times during eukaryotic radiations.

Given that the heterodimeric TALEs evolved in a lineage-specific manner, we asked what the proto-TALE looked like at the time it underwent duplication. The following observations suggest that the proto-TALE was a homodimerizing protein. First, the PBC-homology domains of PBX/GLX class proteins identified in the Archaeplastida includes the MEINOX-motif that was originally defined for its similarity to the MEIS/KNOX-homology domains (Fig. 3) [14]. Second, PBX-Glauco sequences possess the ELK-homology within their PBL domain (Fig. 3), which align well to the ELK domains of KNOX class sequences in Viridiplantae (Additional file 3: Figure S15). Therefore, the MEINOX-motif and ELK-homology across the heterodimerizing KNOX and PBX groups supported the common origin of heterodimerizing TALE groups from a single TALE by duplication followed by subfunctionalization.

## Discussion

### TALE endowment in Archaeplastida

Our study shows that all three Archaeplastida phyla possess TALEs, diverged into two groups with distinct heterodimerization domains, the KNOX group with KN-A/KN-B domains and the PBX (or GLX) group with PBL domains. The similarity between the KNOX/PBX and the animal MEIS/PBC dyads led us to identify homologous heterodimerization domains in the TALEs of other eukaryotic lineages including Excavata. Based on our findings, we hypothesize that the TALE heterodimerization arose very early in eukaryotic evolution.

During > 1 BY of Archaeplastida history, TALE TF networks have undergone three duplication events compared to the simple dyadic TALEs in Glaucophyta. In Viridiplantae, the KNOX class persists as a single member throughout the mostly unicellular Chlorophyta, whereas it duplicated into KNOX1 and KNOX2 in the multicellular Streptophyta [33]. In Rhodophyta, two KNOX classes, KNOX-Red1 and KNOX-Red2 differ in KN-A and KN-B domains, suggesting sub-functionalization. The third duplication event occurred in the non-KNOX group of the Chlorophyta, whose sequences then underwent rapid divergence in their homeodomain and heterodimerization domains, rendering their classification trickier than other classes. Despite this divergence, proteins in one of the two radiations (Class-B and Mam-B) were found to heterodimerize with KNOX homologs, suggesting that these non-KNOX members serve as regulators of KNOX/GLX heterodimers. We summarize our finding in Fig. 1, Additional file 3: Figures S5, S10.

### What would have been the critical drivers of TALE heterodimerization networks emerging from ancestral homodimers?

We found two conserved intron positions and shared sequence motifs between the KNOX- and PBX-groups, generating our hypothesis that a proto-TALE protein initially engaged in homodimerization and then duplicated and diversified into two heterodimerizing classes (Fig. 1, Additional file 3: Figure S5). Heterodimerization-dependent subcellular localization [10, 34], coupled with numerous combinations of distinct DNA-binding modules that fine-tune target specificity, then generated customized transcription-on switches.

During sexual development, it is critical to accurately detect the fusion of two cells before initiating diploid development and to make sure that the mating combines correct partner gametes. TF heterodimerization can implement both steps if one TF partner is contributed by each gamete. In fact, TALE heterodimerization plays a central role as a developmental switch for the haploid-to-diploid transition in green algae and land plants [10, 19]. A similar haploid-to-diploid transition triggered by TF heterodimerization has recently been documented in *Dictyostelium* [35] and is well described in Basidiomycete fungi that utilize non-TALE homeobox proteins such as bW and bE [36, 37].

Discovery of new prokaryotic life forms, especially in the Archaea domain, suggests that multiple symbiotic mergers of different life forms evolved into the proto-eukaryotes, possibly first as a symbiotic community, which then evolved into the last eukaryotic common ancestors (LECA) that rapidly diverged into the eukaryotic supergroups [38–40]. This eukaryogenesis model predicts that the proto-eukaryotes ➔ LECA transition required the faithful transmission of traits between progenitor cells and their progeny to evolve as individual lineages by Darwinian selection. Under this hypothesis, we anticipate that the generation of the LECA may have been driven by the sexual mechanisms that distinguish a cellular merger between the common descendants from a merger between unrelated community members. Our proposal for the evolution of heterodimeric TALEs from the homodimeric proto-TALE may provide one of the necessary mechanisms for the first sexual mode of reproduction that might have driven the generation of the LECA from its proto-eukaryotic ancestors.

### Does expansion of heterodimerizing TALE TFs relate to the emergence of multicellular complexity?

Plant studies have shown that the duplicated KNOX classes serve distinct functions: the plant KNOX1 class regulates the differentiation of an undifferentiated cell mass into spores in mosses or leafy organs in vascular plants, and the plant KNOX2 class regulates the transition from haploid gametophytes to diploid sporophytes in mosses and controls secondary cell wall development in vascular plants [18, 41–43]. On the other hand, we know very little about the function of the class B TALE in Viridiplantae and how the heterodimerization network of TALE proteins was restructured following the KNOX1/KNOX2 duplication in Streptophyta. Based on the diversified functions of the KNOX1/KNOX2, we propose that the duplicated TALE heterodimers in the Streptophyta allowed independent regulation of cellular differentiation and life cycle transitions, priming the emergence of land plants by expanding the diploid phase of their life cycle from a dormant zygospore to a multicellular individual bearing many meiotic spores. One of the critical events coinciding the emergence of land plants was the disappearance of the diverse non-KNOX classes except the BELL class, which might have allowed the transition to the multicellular diploid phase by averting the sporogenesis in the zygote. During land plant evolution, the repertoire of TALE heterodimers continued to expand, serving all the major organ differentiation programs in the diploid phase of their life cycle.

Can a similar expansion of TALE heterodimers be found during Metazoan evolution? Our search for TALE TFs in unicellular relatives of the Metazoa—Salpingoeca and Monosiga—revealed a simple configuration with one MEIS- and one PBC-like TALE (Additional file 3: Figures S11, S12), whereas at the Metazoan base one finds at least three MEIS-related classes and two PBC-related classes [44]. These findings suggest the occurrence of a similar expansion of a founding dyad during Metazoan evolution. Therefore, in both plants and animals, the TALE TF network seems to be redeployed for complex multicellularity, departing from its posited original function in sexual development.

Our results suggest that TALE TF networks represent early-evolving developmental mechanisms. That said, the emergence of complex multicellularity doubtless required more than TF networks. TF-based developmental cues need to be propagated via chromatin-level regulatory mechanisms that establish the cellular memory during embryo development. The extent to which chromatin-level regulatory mechanisms are involved in the development of unicellular organisms is a critical question in elucidating the origins of complex multicellularity.

## Conclusions

Our study explored a deeper evolutionary history of heterodimerizing TALE transcription factors and identified true homology among the protein domains that mediate the TALE heterodimerization of the animal MEIS/PBC dyads and the algal KNOX/GSP1 dyads. We showed that the homology extends to Excavata lineage close to the last eukaryotic common ancestors. Collectively, our findings place the origin of TALE-TALE heterodimerization near the eukaryotic root. Considering profoundly conserved sexual role of the TALE heterodimer in Viridiplantae [10, 18, 19], we hypothesize that the TALE heterodimeric configuration evolved to provide a means to ensure whether the cell fusion is correctly executed between appropriate partner gametes as a prerequisite for the evolution of eukaryotic sexuality.

## Methods

### Strains and culture conditions

Axenic *Micromonas commoda* (RCC299) and *Ostreococcus tauri* (OTH95) were maintained in Keller medium [45] in artificial seawater at room temperature. One hundred mL of a 14-day-old culture was harvested for genomic DNA extraction. *Picocystis salinarum* (CCMP1897) was obtained from the National Center for Marine Algae and Microbiota (NCMA), maintained in L1 medium [46] in artificial sea water, and plated on 1.5% Bactoagar-containing media for single-colony isolation. Genomic DNA of *P. salinarum* was then obtained from a culture derived from one colony.

### Phylogenetic analysis and classification of homeobox genes

Archaeplastida algal TALE homeodomains were collected from the available genomes and transcriptomes listed in Additional file 1: Table S1. Details of how TALE sequence was collected is provided in Additional file 5: Method S1. After excluding nearly identical sequences, a total of 96 sequences together with 18 reference TALE sequences were made into the final homeodomain alignment with 70 unambiguously aligned positions with eight gapped and one constant sites. Details of phylogenetic reconstruction is provided in Additional file 5: Method S2.

### Bioinformatics analysis

The entire TALE collection was divided into multiple groups representing major clades in the homeodomain tree. Each group was individually analyzed by running MEME4.12 in the motif-discovery mode with default option collecting up to 10 motifs at http://meme-suite.org/ [47]. The search provided multiple non-overlapping motifs, many of which were combined according to previously identified domains such as bipartite KN-A/KN-B, ELK, and HD [14] and independent domain searches against the INTERPRO database (http://www.ebi.ac.uk/interpro/) [48]. All the collected TALE-associated homology domains were aligned to generate HMM motifs by HMMbuild (v3.1b2), which we used to test if these homology domains are specific to the TALE sequences using HMMsearch (v3.1b2) against the genome-wide protein collections with E-value of 0.01 as the per-domain inclusion threshold [49]. All the homology domain information was used to locate any error in gene predictions, and gene models were updated if necessary (Details of the gene model curation is provided in Additional file 5: Method S3).

### Intron comparison

Introns within the homeodomain were collected and labeled as site numbers of the homeodomain (1–60 plus 'abc' for the three amino acid extension shared by the TALE homeodomain). If an intron is between two codons it is denoted N/N + 1, where N is the last amino acid site number of the preceding exon; introns within a codon are denoted N(n/n + 1), where n is one or two for the codon nucleotide position relative to the splice-sites.

### Yeast-two-hybrid analysis

*M. commoda* (affixed with Micco), *O. tauri* (affixed with Ostta), and *P. salinarum* (affixed with Picsa) TALE protein coding sequences were cloned by PCR using primers designed herein (Additional file 1) from genomic DNAs prepared by the phenol/chloroform extraction and ethanol precipitation method. Micco_62153 and Picsa_04684 contained a single intron, whereas all the other nine genes lacked an intron in the entire open reading frame. For cloning of Micco_62153, we synthesized the middle fragment lacking the intron and ligated them via *Xho*I and *Cla*I sites. For cloning details, see Additional file 5: Method S4.

## Additional files

Additional file 1: **Table S1.** Genomic resources used in this study. **Table S2**. Archaeplastidal homeobox collection of TALE protein analyzed in this study. **Table S3**. KNOX domain homology among KNOX classes. **Table S4**. Primers used in this study. **Table S5**. Yeast-two-hybrid constructs used in this study. **Table S6**. Homeobox profile in Trebouxiophyceae. (XLSX 370 kb)

Additional file 2: **Note S1.** Lack of TALE TFs in Trebouxiophyceae. **Note S2**. Horizontal transfer may explain the presence of Rhodophyta TALE heterodimers in *Picocystis and Klebsormidium* of Viridiplantae. (PDF 55 kb)

Additional file 3: **Figure S1.** Alignment of TALE homeodomain sequences of the Archae-algal collection. **Figure S2**. Homology domain alignment of KNOX homologs. **Figure S3**. GLX class is defined by PBL-Chloro domain. **Figure S4**. Extensive homology of Picsa_04995 and Klefl_00021_0250 to Chocr_41034 indicates their classification as PBX-Red. **Figure S5**. Intron-retention pattern suggests parallel evolution of KNOX and non-KNOX group classes from common duplicated TALE ancestors. **Figure S6**. Alignment of Class-B TALE proteins in volvocales. **Figure S7**. Alignment of Mam-A TALE proteins in mamiellophyceae. **Figure S8**. Alignment of Mam-B TALE proteins in mamiellophyceae. **Figure S9**. Full-length proteins are necessary for mamiellophyceae non-KNOX TALE proteins to form heterodimers. **Figure S10**. TALE interaction network defined by this study using yeast-two-hybrid assays. **Figure S11**. Identification of MEIS homologs in choanoflagellates. **Figure S12**. Identification of PBX homologs in choanoflagellates. **Figure S13**. Alignment of the MEIS homologs in Amorphea. **Figure S14**. Alignment of the PBX homologs in Amorphea. **Figure S15**. ELK-domain alignment. (PDF 10098 kb)

Additional file 4: **Data S1.** Results of HMM-motif search for KN-A, KN-B, and PBL-Red domains. **Data S2**. Alignment of the KN-A, KN-B, and PBL-Red domains. (PDF 66 kb)

Additional file 5: **Method S1.** Collecting TALE homeobox protein sequences. **Method S2**. Phylogenetic reconstruction. **Method S3**. Homology motif/domain search. **Method S4**. Intron comparison. **Method S5**. Cloning of Yeast-two-hybrid constructs. (PDF 57 kb)

## Abbreviations

GLX: GSP1-like homeobox
LECA: Last eukaryotic common ancestors
PBL: PBC-B like
proto-TALE: Prototypical TALE
TALE: Three amino acid length extension
TF: Transcription factor

## References

[1] Billeter M, Qian YQ, Otting G, Muller M, Gehring W, Wuthrich K (1993). Determination of the nuclear magnetic resonance solution structure of an Antennapedia homeodomain-DNA complex. J Mol Biol.

[2] Azpiazu N, Morata G (1998). Functional and regulatory interactions between Hox and extradenticle genes. Genes Dev.

[3] Hudry B, Thomas-Chollier M, Volovik Y, Duffraisse M, Dard A, Frank D (2014). Molecular insights into the origin of the Hox-TALE patterning system. elife.

[4] Hake S, Smith HMS, Holtan H, Magnani E, Mele G, Ramirez J (2004). The role of KNOX genes in plant development. Annu Rev Cell Dev Biol.

[5] Hay A, Tsiantis M (2010). KNOX genes: versatile regulators of plant development and diversity. Development.

[6] Berthelsen J, Kilstrup-Nielsen C, Blasi F, Mavilio F, Zappavigna V (1999). The sub cellular localization of PBX1 and EXD proteins depends on nuclear import and export signals and is modulated by association with PREP1 and HTH. Genes Dev.

[7] Stevens KE, Mann RS (2007). A balance between two nuclear localization sequences and a nuclear export sequence governs extradenticle subcellular localization. Genetics.

[8] Bhatt AM, Etchells JP, Canales C, Lagodienko A, Dickinson H (2004). VAAMANA--a BEL1-like homeodomain protein, interacts with KNOX proteins BP and STM and regulates inflorescence stem growth in Arabidopsis. Gene.

[9] Smith HMS, Hake S (2003). The interaction of two homeobox genes, BREVIPEDICELLUS and PENNYWISE, regulates internode patterning in the Arabidopsis inflorescence. Plant Cell.

[10] Lee J-H, Lin H, Joo S, Goodenough U (2008). Early sexual origins of homeoprotein heterodimerization and evolution of the plant KNOX/BELL family. Cell.

[11] Noyes MB, Christensen RG, Wakabayashi A, Stormo GD, Brodsky MH, Wolfe SA (2008). Analysis of homeodomain specificities allows the family-wide prediction of preferred recognition sites. Cell.

[12] Bürglin TR (1997). Analysis of TALE superclass homeobox genes (MEIS, PBC, KNOX, Iroquois, TGIF) reveals a novel domain conserved between plants and animals. Nucleic Acids Res.

[13] Bellaoui M, Pidkowich MS, Samach A, Kushalappa K, Kohalmi SE, Modrusan Z (2001). The Arabidopsis BELL1 and KNOX TALE homeodomain proteins interact through a domain conserved between plants and animals. Plant Cell.

[14] Bürglin TR (1998). The PBC domain contains a MEINOX domain: coevolution of Hox and TALE homeobox genes?. Dev Genes Evol.

[15] Mukherjee K, Brocchieri L, Bürglin TR (2009). A comprehensive classification and evolutionary analysis of plant homeobox genes. Mol Biol Evol.

[16] Nishimura Y, Shikanai T, Nakamura S, Kawai-Yamada M, Uchimiya H (2012). Gsp1 triggers the sexual developmental program including inheritance of chloroplast DNA and mitochondrial DNA in Chlamydomonas reinhardtii. Plant Cell.

[17] Joo S, Nishimura Y, Cronmiller E, Hong RH, Kariyawasam T, Wang MH (2017). Gene regulatory networks for the haploid-to-diploid transition of Chlamydomonas reinhardtii. Plant Physiol.

[18] Sakakibara K, Ando S, Yip HK, Tamada Y, Hiwatashi Y, Murata T (2013). KNOX2 genes regulate the haploid-to-diploid morphological transition in land plants. Science.

[19] Horst NA, Katz A, Pereman I, Decker EL, Ohad N, Reski R (2016). A single homeobox gene triggers phase transition, embryogenesis and asexual reproduction. Nat Plants.

[20] Adl SM, Simpson AGB, Lane CE, Lukeš J, Bass D, Bowser SS (2012). The revised classification of eukaryotes. J Eukaryot Microbiol.

[21] Worden AZ, Follows MJ, Giovannoni SJ, Wilken S, Zimmerman AE, Keeling PJ (2015). Rethinking the marine carbon cycle: factoring in the multifarious lifestyles of microbes. Science.

[22] Guillou Laure, Eikrem Wenche, Chrétiennot-Dinet Marie-Josèphe, Le Gall Florence, Massana Ramon, Romari Khadidja, Pedrós-Alió Carlos, Vaulot Daniel (2004). Diversity of Picoplanktonic Prasinophytes Assessed by Direct Nuclear SSU rDNA Sequencing of Environmental Samples and Novel Isolates Retrieved from Oceanic and Coastal Marine Ecosystems. Protist.

[23] Lewis LA, McCourt RM (2004). Green algae and the origin of land plants. Am J Bot.

[24] Yoon HS, Muller KM, Sheath RG, Ott FD, Bhattacharya D (2006). Defining the major lineages of red algae (RHODOPHYTA). J Phycol.

[25] Jackson C, Clayden S, Reyes-Prieto A (2015). The Glaucophyta: the blue-green plants in a nutshell. Acta Soc Bot Pol.

[26] Worden AZ, Lee JH, Mock T, Rouze P, Simmons MP, Aerts AL (2009). Green evolution and dynamic adaptations revealed by genomes of the marine Picoeukaryotes Micromonas. Science.

[27] Archibald JM (2015). Genomic perspectives on the birth and spread of plastids. Proc Natl Acad Sci.

[28] Bertolino E, Reimund B, Wildt-Perinic D, Clerc RG (1995). A novel homeobox protein which recognizes a TGT core and functionally interferes with a retinoid-responsive motif. J Biol Chem.

[29] Kurvari V, Grishin NV, Snell WJ (1998). A gamete-specific, sex-limited homeodomain protein in Chlamydomonas. J Cell Biol.

[30] Piganeau G, Grimsley N, Moreau H (2011). Genome diversity in the smallest marine photosynthetic eukaryotes. Res Microbiol.

[31] Clarke M, Lohan AJ, Liu B, Lagkouvardos I, Roy S, Zafar N (2013). Genome of Acanthamoeba castellanii highlights extensive lateral gene transfer and early evolution of tyrosine kinase signaling. Genome Biol.

[32] Sverdlov AV, Rogozin IB, Babenko VN, Koonin EV (2005). Conservation versus parallel gains in intron evolution. Nucleic Acids Res.

[33] Frangedakis E, Saint-Marcoux D, Moody LA, Rabbinowitsch E, Langdale JA (2016). Nonreciprocal complementation of KNOX gene function in land plants. New Phytol.

[34] Longobardi E, Penkov D, Mateos D, De Florian G, Torres M, Blasi F. Biochemistry of the tale transcription factors PREP, MEIS, and PBX in vertebrates. Wellik D, Torres M, Ros M, editors Dev Dyn 2014;243: 59–75. 10.1002/dvdy.24016 PMC4232920 23873833

[35] Hedgethorne K, Eustermann S, Yang J-C, Ogden TEH, Neuhaus D, Bloomfield G. Homeodomain-like DNA binding proteins control the haploid-to-diploid transition in Dictyostelium. Sci Adv. 2017;3:e1602937. 10.1126/sciadv.1602937 PMC5580921 28879231

[36] Kües U, Asante-Owusu RN, Mutasa ES, Tymon AM, Pardo EH, O'Shea SF (1994). Two classes of homeodomain proteins specify the multiple a mating types of the mushroom Coprinus cinereus. Plant Cell.

[37] Spit A, Hyland RH, Mellor EJC, Casselton LA (1998). A role for heterodimerization in nuclear localization of a homeodomain protein. Proc Natl Acad Sci U S A.

[38] O’Malley MA (2015). Endosymbiosis and its implications for evolutionary theory. Proc Natl Acad Sci U S A.

[39] López-García P, Eme L, Moreira D (2017). Symbiosis in eukaryotic evolution. J Theor Biol.

[40] Zaremba-Niedzwiedzka K, Caceres EF, Saw JH, Bäckström D, Juzokaite L, Vancaester E (2017). Asgard archaea illuminate the origin of eukaryotic cellular complexity. Nature.

[41] Barton MK, Poethig RS (1993). Formation of the shoot apical meristem in *Arabidopsis thaliana*: an analysis of development in the wild type and in the shoot meristemless mutant. Development.

[42] Li E, Bhargava A, Qiang W, Friedmann MC, Forneris N, Savidge RA (2012). The class II KNOX gene KNAT7 negatively regulates secondary wall formation in Arabidopsis and is functionally conserved in Populus. New Phytol.

[43] Furumizu Chihiro, Alvarez John Paul, Sakakibara Keiko, Bowman John L. (2015). Antagonistic Roles for KNOX1 and KNOX2 Genes in Patterning the Land Plant Body Plan Following an Ancient Gene Duplication. PLOS Genetics.

[44] Mukherjee K, Bürglin TR (2007). Comprehensive analysis of animal TALE homeobox genes: new conserved motifs and cases of accelerated evolution. J Mol Evol.

[45] Keller MD, Selvin RC, Claus W, Guillard RRL (2007). Media for the culture of oceanic ultraphytoplankton1,2. J Phycol.

[46] Guillard R. R. L., Hargraves P. E. (1993). Stichochrysis immobilis is a diatom, not a chrysophyte. Phycologia.

[47] Bailey TL, Johnson J, Grant CE, Noble WS (2015). The MEME suite. Nucleic Acids Res.

[48] Finn RD, Attwood TK, Babbitt PC, Bateman A, Bork P, Bridge AJ (2017). InterPro in 2017-beyond protein family and domain annotations. Nucleic Acids Res.

[49] HMMER: biosequence analysis using profile hidden Markov models. 2017. http://www.hmmer.org. Accessed 3 June 2017.

