# Correction to: Common ancestry of heterodimerizing TALE homeobox transcription factors across Metazoa and Archaeplastida

**DOI:** 10.1186/s12915-020-0737-2

**Published:** 2020-01-09

**Authors:** Sunjoo Joo, Ming Hsiu Wang, Gary Lui, Jenny Lee, Andrew Barnas, Eunsoo Kim, Sebastian Sudek, Alexandra Z. Worden, Jae-Hyeok Lee

**Affiliations:** 1 Department of Botany, University of British Columbia, 6270 University Blvd, Vancouver, BC V6T 1Z4 Canada; 2 Division of Invertebrate Zoology and Sackler Institute for Comparative Genomics, American Museum of Natural History, 200 Central Park West, New York, NY 10024 USA; 3 Monterey Bay Aquarium Research Institute, 7700 Sandholdt Rd, Moss Landing, CA 95039 USA; 4 Ocean EcoSystems Biology Unit, RD3 GEOMAR Helmholtz Centre for Ocean Research Kiel, Kiel, Germany

**Correction to: BMC Biology**

**https://doi.org/10.1186/s12915-018-0605-5**

Upon publication of the original article [1], it was noticed that Alexandra Z. Worden’s affiliation is not complete. The full affiliation information for Alexandra Z. Worden can be found below and in the complete affiliation list of this Correction article.

- Ocean EcoSystems Biology Unit, RD3 GEOMAR Helmholtz Centre for Ocean Research Kiel, Kiel, Germany.
- Monterey Bay Aquarium Research Institute, 7700 Sandholdt Rd., Moss Landing, CA 95039, USA.
