# Supplementary material for: Common ancestry of heterodimerizing TALE homeobox transcription factors across Metazoa and Archaeplastida
Source: BMC Biol. 2018 Nov 5;16:136. doi: 10.1186/s12915-018-0605-5 (PMC6219170; doi:10.1186/s12915-018-0605-5)
Supplement: Supplementary file 2 — Note S1. Lack of TALE TFs in Trebouxiophyceae. Note S2. Horizontal transfer may explain the presence of Rhodophyta TALE heterodimers in Picocystis and Klebsormidium of Viridiplantae. (PDF 55 kb) [file 12915_2018_605_MOESM2_ESM.pdf]

Additional file 2 for Joo et al.

Article title: Evolution of heterodimerizing TALE homeobox transcription factors as a developmental mechanism for the haploid-to-diploid transition.

Authors: Sunjoo Joo, Ming Hsiu Wang, Gary Lui, Jenny Lee, Andrew Barnas, Eunsoo Kim, Sebastian Sudek, Alexandra Z. Worden, and Jae-Hyeok Lee.

Supplemental Notes

S1. Lack of TALE TFs in Trebouxiophyceae

Trebouxiophyceae is one of the four major radiations in Chlorophyta, and presents an exception to the universal presence of TALEs in Archaeplastida, where five annotated genomes are available from *Chlorella* sp. NC64A, *Chlorella protothecoides*, *Asterochloris*, *Coccomyxa* C-169, and *Helicosporidium* sp. (Blanc et al., 2010; Gao et al., 2014; Blanc et al., 2012; Foflonker et al., 2015, Pombert et al., 2014). Additional searches in seven other draft genomes did not recover TALE sequences (S1 Spreadsheet), albeit other homeobox genes were well conserved throughout the Trebouxiophycean species, which are grouped into HOXDDT, OCP3, HDZIP, and WOX classes that are reported in Chlorophyta (Wilhelmsson et al., 2017) (S6 Spreadsheet). Hence all TALE genes were apparently lost early after the divergence of Trebouxiophyceae from other Chlorophyta algae.

S2. Horizontal transfer may explain the presence of Rhodophyta TALE heterodimers in *Picocystis and Klebsormidium* of Viridiplantae

Most Chlorophyta species possess one KNOX and two non-KNOX TALE sequences, but *P.salinarum* (CCMP1897) of the prasinophyte class VII, also known as *Chloropicophyceae* (Lopes Dos Santos et al., 2017), is exceptional, having two additional homeobox sequences of possible red algal origin in addition to the usual three TALE sequences (asterisks in Fig 2): Picsa_0325 has a KNOX-Red2-specific KN-C2 domain, and Picsa_04995 shows overall 24% identity to the red alga *Chondrus crispus* PBX-Red homolog (Chocr_41034) in its PBX domain and its upstream sequence (S4 Fig). We excluded the possibility of red-algal contamination in the analyzed MMETSP transcriptome by successful cloning of all five TALE sequences from our own DNA preps from single cell-derived colonies made from an axenic *Picocystis* CCMP1897 culture. In a second exception, the charophyte *K. flaccidum* genome possesses six TALE sequences classified into distinct clades (plus signs in Fig 2), which include Klefl_00157_0150 with KN-C2 and Klefl_00021_0250 which shows overall 16% identity to Chocr_41034 (S4 Fig). We propose that in both cases, these sequences may have been obtained from a red algal vector via horizontal transfer, which would have entailed the co-transfer of heterodimerizing TFs

in two parallel events. KNOX-Red2 sequences are highly conserved, suggesting their recent origin in Rhodophyta followed by horizontal transfer to certain Viridiplantae species.

We asked if the red-algal TALEs interact with the green-algal ones coexisting in *P. salinarum*. The KNOX-Red2 homolog (Picsa_0325) was found to interact with the two non-KNOX TALEs carrying different PBL domains but not with the non-KNOX without it (Class-B) (S10B Fig), indicating that heterodimerization entails an interaction between KN-A/KN-B and PBL domains. On the other hand, the KNOX-Chloro homolog (Picsa_04387) interacts with the GLX and Class-B sequences, but not with the PBX-Red homolog containing a PBL domain (S10B Fig). This suggests that KNOX-Chloro proteins may have evolved an ability to distinguish multiple versions of PBL domains. In contrast, the KNOX-Red2 homolog displays broad-specificity interactions, reflecting its original Rhodophyta TALE configuration where only one PBX-Red class protein is present in the TALE heterodimerization network.

References in the supporting information

Biasini M, Bienert S, Waterhouse A, Arnold K, Studer G, Schmidt T, Kiefer F, Gallo Cassarino T, Bertoni M, Bordoli L, et al. 2014. SWISS-MODEL: modelling protein tertiary and quaternary structure using evolutionary information. Nucleic Acids Research 42: W252–8.

Blanc G, Agarkova I, Grimwood J, Kuo A, Brueggeman A, Dunigan DD, Gurnon J, Ladunga I, Lindquist E, Lucas S, et al. 2012. The genome of the polar eukaryotic microalga Coccomyxa subellipsoidea reveals traits of cold adaptation (J Sullivan, Ed.). Genome Biology 13: R39–552.

Blanc G, Duncan G, Agarkova I, Borodovsky M, Gurnon J, Kuo A, Lindquist E, Lucas S, Pangilinan J, Polle J, et al. 2010. The Chlorella variabilis NC64A genome reveals adaptation to photosymbiosis, coevolution with viruses, and cryptic sex. THE PLANT CELL ONLINE 22: 2943–2955.

Bürglin TR. 1997. Analysis of TALE superclass homeobox genes (MEIS, PBC, KNOX, Iroquois, TGIF) reveals a novel domain conserved between plants and animals. Nucleic Acids Research 25: 4173–4180.

Foflonker F, Price DC, Qiu H, Palenik B, Wang S, Bhattacharya D. 2015. Genome of the halotolerant green alga Picochlorum sp. reveals strategies for thriving under fluctuating environmental conditions. (JE Hallsworth, Ed.). Environmental Microbiology 17: 412–426.

Gao C, Wang Y, Shen Y, Yan D, He X, Dai J, Wu Q. 2014. Oil accumulation mechanisms of the oleaginous microalga Chlorella protothecoides revealed through its genome, transcriptomes, and proteomes. BMC genomics 15: 582.

Guillou, Eikrem, Massana, Romari, Vaulot. 2004. Diversity of Picoplanktonic Prasinophytes Assessed by Direct Nuclear SSU rDNA Sequencing of Environmental Samples and Novel Isolates Retrieved from Oceanic and Coastal Marine Ecosystems. Annals of Anatomy 155: 22–22.

Guindon S, Dufayard J-F, Lefort V, Anisimova M, Hordijk W, Gascuel O. 2010. New algorithms and methods to estimate maximum-likelihood phylogenies: assessing the performance of PhyML 3.0. Systematic biology 59: 307–321.

Jackson C, Clayden S, Reyes-Prieto A. 2015. The Glaucophyta: the blue-green plants in a nutshell. Acta Societatis Botanicorum Poloniae 84: 149–165.

Keeling PJ, Burki F, Wilcox HM, Allam B, Allen EE, Amaral-Zettler LA, Armbrust EV, Archibald JM, Bharti AK, Bell CJ, et al. 2014. The Marine Microbial Eukaryote Transcriptome Sequencing Project (MMETSP): illuminating the functional diversity of eukaryotic life in the oceans through transcriptome sequencing. (RG Roberts, Ed.). PLoS Biol 12: e1001889.

Lopes Dos Santos A, Pollina T, Gourvil P, Corre E, Marie D, Garrido JL, Rodríguez F, Noël M-H, Vaulot D, Eikrem W. 2017. Chloropicophyceae, a new class of picophytoplanktonic prasinophytes. Scientific Reports 7: 14019.

Luo A, Qiao H, Zhang Y, Shi W, Ho SY, Xu W, Zhang A, Zhu C. 2010. Performance of criteria for selecting evolutionary models in phylogenetics: a comprehensive study based on simulated datasets. BMC evolutionary biology 10: 242.

Minh BQ, Nguyen MAT, Haeseler von A. 2013. Ultrafast approximation for phylogenetic bootstrap. Molecular Biology and Evolution 30: 1188–1195.

Rokas A. 2011. Phylogenetic analysis of protein sequence data using the Randomized Axelerated Maximum Likelihood (RAXML) Program. (FM Ausubel, R Brent, RE Kingston, DD Moore, JG Seidman, JA Smith, and K Struhl, Eds.). Current protocols in molecular biology Chapter 19: Unit19.11–19.11.14.

Ronquist F, Teslenko M, van der Mark P, Ayres DL, Darling A, Höhna S, Larget B, Liu L, Suchard MA, Huelsenbeck JP. 2012. MrBayes 3.2: efficient Bayesian phylogenetic inference and model choice across a large model space. Systematic biology 61: 539–542.

Wilhelmsson PKI, Mühlich C, Ullrich KK, Rensing SA. 2017. Comprehensive Genome-Wide Classification Reveals That Many Plant-Specific Transcription Factors Evolved in Streptophyte Algae. Genome Biology and Evolution 9: 3384–3397.
