# Supplementary material for: Common ancestry of heterodimerizing TALE homeobox transcription factors across Metazoa and Archaeplastida
Source: BMC Biol. 2018 Nov 5;16:136. doi: 10.1186/s12915-018-0605-5 (PMC6219170; doi:10.1186/s12915-018-0605-5)

Additional files 3 for Joo et al.

Article title: Common ancestry of heterodimerizing TALE homeobox transcription factors across Metazoa and Archaeplastida.

Authors: Sunjoo Joo, Ming Hsiu Wang, Gary Lui, Jenny Lee, Andrew Barnas, Eunsoo Kim, Sebastian Sudek, Alexandra Z. Worden, and Jae-Hyeok Lee.

The following Supporting Information is available for this article:

Fig. S1. Alignment of TALE homeodomain sequences of the Archae-algal collection.

Fig. S2. Homology domain alignment of KNOX sequences.

Fig. S3. GLX class is defined by PBL-Chloro domain.

Fig. S4. Extensive homology of Picsa_04995 and Klefl_00021_0250 to Chocr_41034 indicates their classification as PBX-Red.

Fig. S5. Intron-retention pattern suggests parallel evolution of KNOX and non-KNOX group classes from common duplicated TALE ancestors.

Fig. S6. Alignment of Class-B TALE proteins in volvocales.

Fig. S7. Alignment of Mam-A TALE proteins in mamiellophyceae.

Fig. S8. Alignment of Mam-B TALE proteins in mamiellophyceae.

Fig. S9. Full-length proteins are necessary for mamiellophyceae non-KNOX TALE proteins to form heterodimers.

Fig. S10. TALE interaction network defined by this study using yeast-two-hybrid assays.

Fig. S11. Identification of MEIS homologs in choanoflagellates.

Fig. S12. Identification of PBX homologs in choanoflagellates.

Fig. S13. Alignment of the MEIS homologs in Amorphea.

Fig. S14. Alignment of the PBX homologs in Amorphea.

Fig. S15. ELK-domain alignment.

Fig. S1. Alignment of TALE homeodomain sequences of the Archaeplastida-algal collection. The 106 sequences were made into an alignment after excluding 20 near identical sequences to reduce redundancy. Animal/amoeba/haptophyte outgroup sequences are included as they share homology with Archaeplastida TALEs outside the homeodomain. The three bars above the sequence numbers show predicted alpha helices. Discarded insertions are noted in red arrowheads.

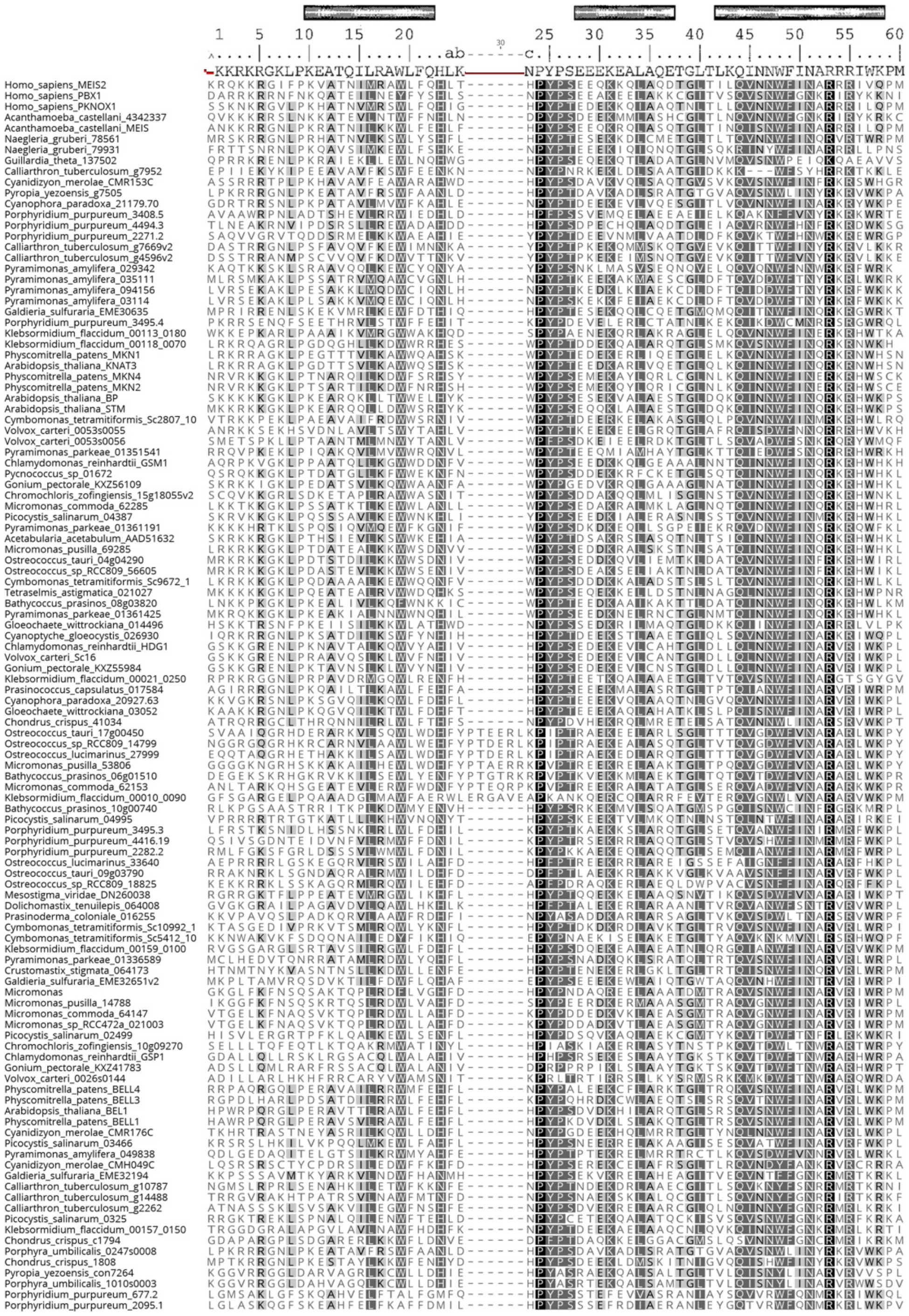

Fig. S2. Homology domain alignment of KNOX sequences. MEIS class outgroup sequences are included at the bottom. Class label is on the left. KN-A, KN-B, ELK, HOMEOBOX, KN-C1, and KN-C2 domains are labeled on the top. Class groups are labeled by colored bars on the left next to the gene names. Yellow, light green, and green shades in sequences show more than 60%, 80%, or 100% similarity in each column. Gaps between KN-A and KN-B and between KN-B and ELK have been eliminated.

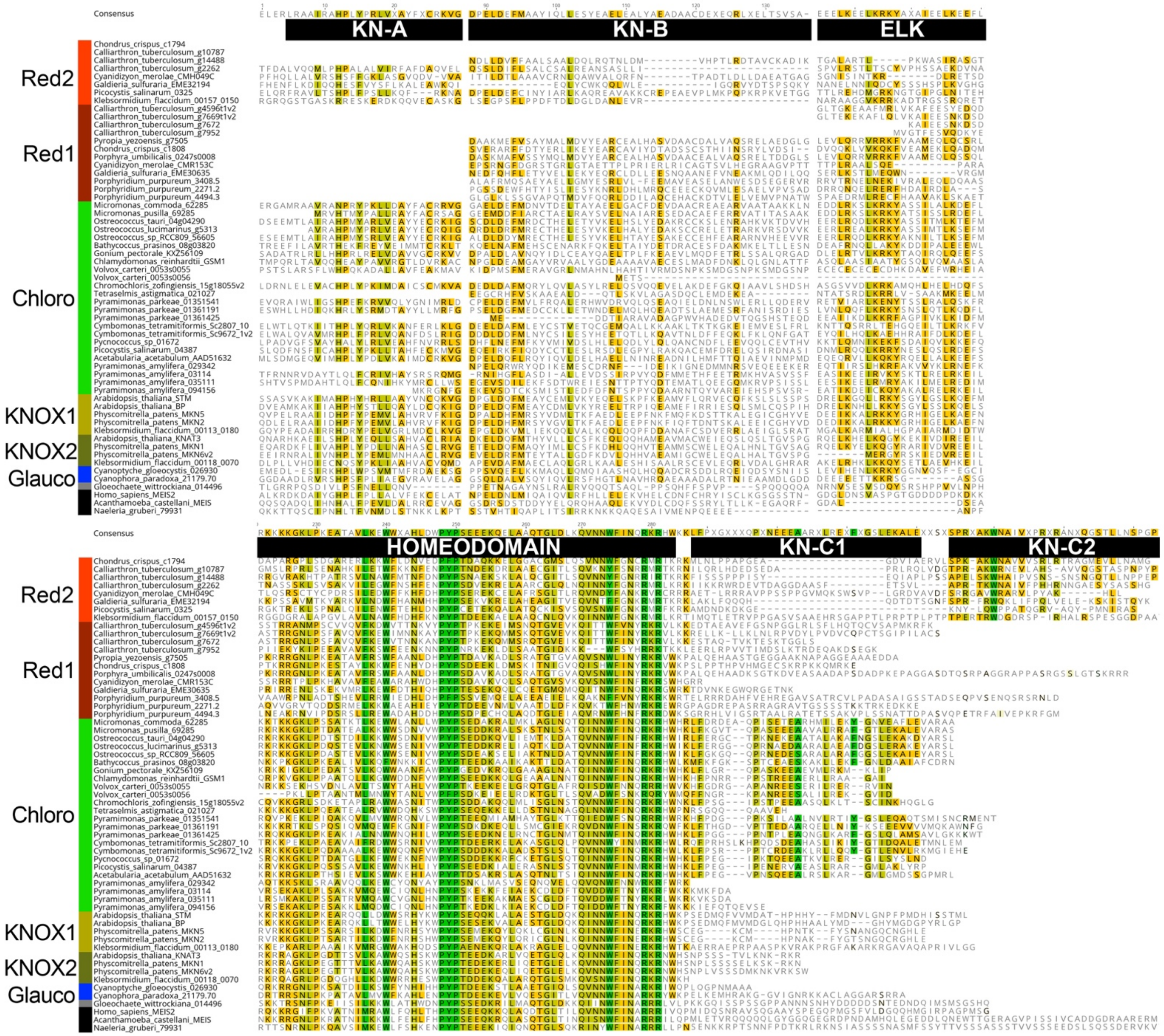

Fig. S3. GLX class is defined by PBL-Chloro domain. (A-C) Three alignments are adjusted with inserting gaps for direct comparison among different PBL-Chloro domains. (A) GLX-Chloro class members. (B) GLX-Basal class members. (C) Three Viridiplantae sequences with strong MEINOX homology domain. PBC-homology is shared among the Chlorophyta non-KNOX sequences.

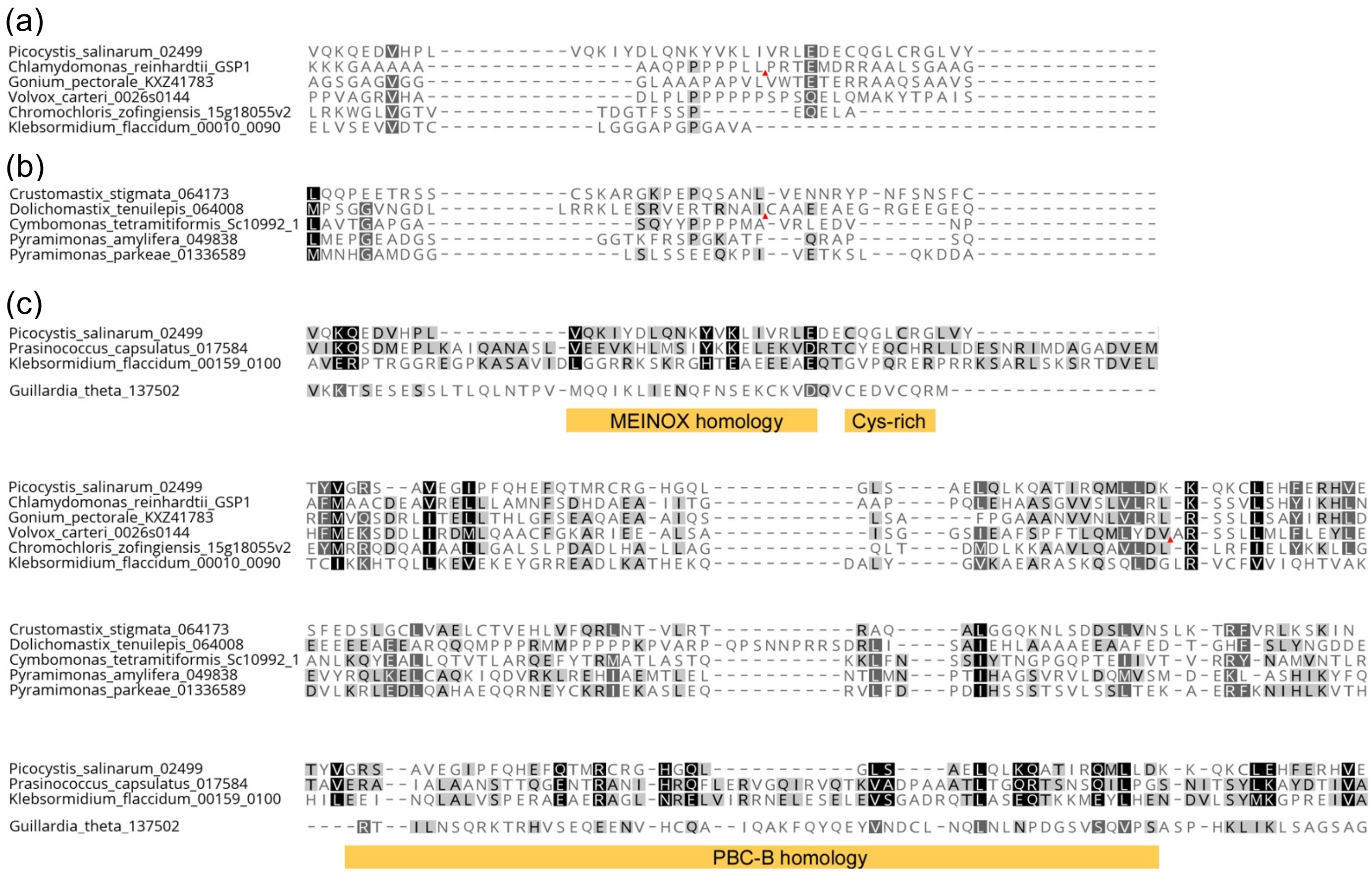

Fig. S4. Extensive homology of Picsa_04995 and Klefl_00021_0250 to Chocr_41034 indicates their classification as PBX-Red. MEINOX-homology and PBL-Red domains are indicated by red bars below the alignment.

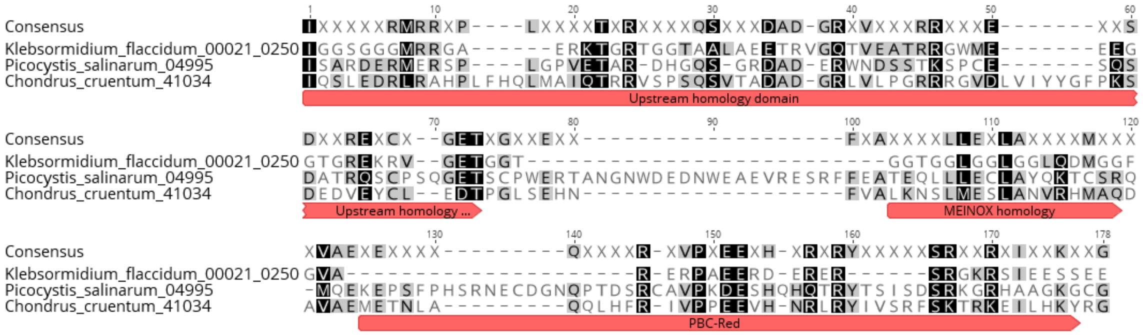

Fig. S5. Intron-retention pattern suggests parallel evolution of KNOX and non-KNOX group classes from common duplicated TALE ancestors. (A) Intron locations collected from 12 TALE classes are shown with arrows. Half arrows indicate cases where not all the class members share the position. White arrows indicate shared positions in at least two different classes, and black arrows indicate class-specific positions. The numbers above the consensus sequence show 60 amino acid positions; the three-amino-acid extension is denoted as 'abc.' Row color depicts two alternative domain configurations: purple for MEIS/KNOX types, and navy for PBX/GLX types. Class names are colored according to their phylogenetic groups: green for Viridiplantae, red for Rhodophyta, blue for Glaucophyta, and black for outgroups. The numbers following the class names show how many genes provided the intron information. Of the shared positions, purple triangles on the top mark those shared between MEIS/KNOX and PBX/GLX classes, blue triangles mark those shared between GLX and BELL classes, and red triangles mark those shared between KNOX classes. A notable exception is the KNOX-Red1 class, for which three Rhodophyta clades show different intron locations (44/45, 48[2/3] or 53[2/3]), indicating that the 44/45 intron position can indeed be displaced to 48[2/3] or elsewhere, albeit infrequently. The unique 46/47 intron in the PBX-Glauco (Cyapa_20927) would presumably have resulted from a similar displacement in intron position. (B) Distribution of conserved introns among the TALE homeobox classes. Identified TALE classes are mapped on the Arachaeplastida phylogeny. The 44/45 intron is marked by blue outline and the 48[2/3] intron is marked by red outline. Underlines of the class names indicate the presence of a PBC-homology domain. The Archaeplastida phylogeny is modified from figure 1 of Jackson et al. (2015).

(a)

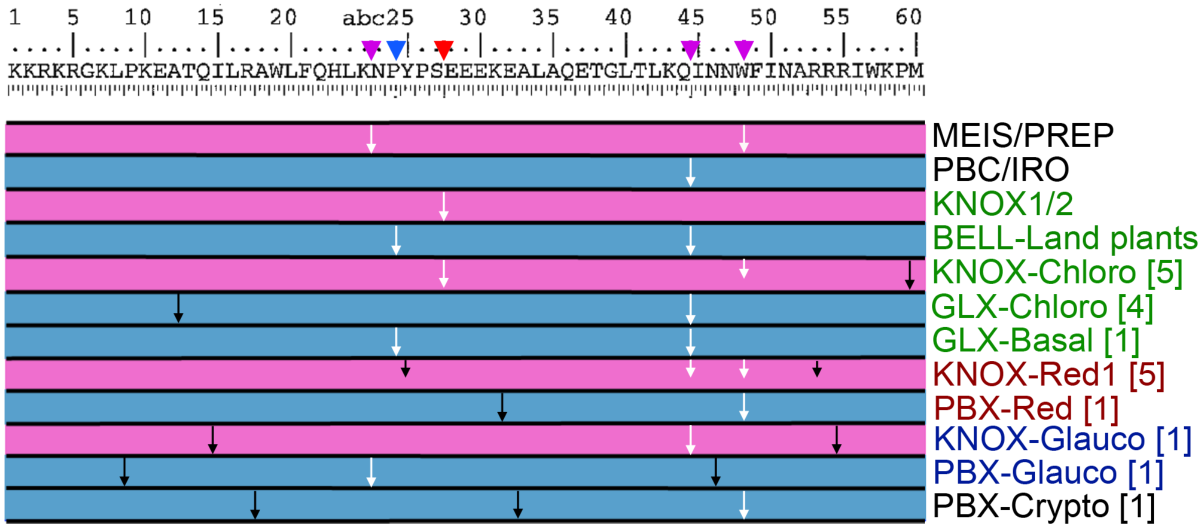

(b)

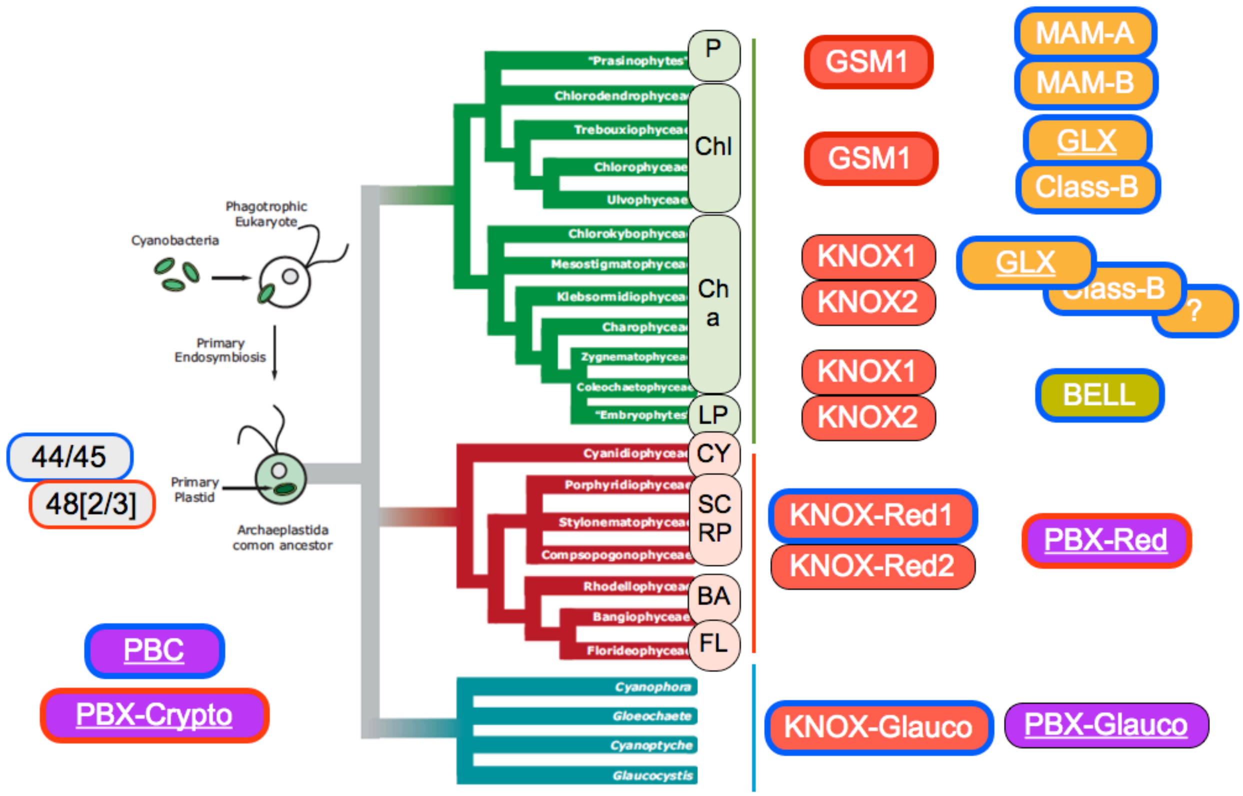

"Prasinophytes"
Chlorodendrophyceae
Trebouxiophyceae
Chlorophyceae
Ulvophyceae
Chlorokybophyceae
Mesostigmatophyceae
Klebsormidiophyceae
Charophyceae
Zygnematophyceae
Coleochaetophyceae
"Embryophytes"
Cyanidiophyceae
Porphyridiophyceae
Stylonematophyceae
Compsopogonophyceae
Rhodellophyceae
Bangiophyceae
Florideophyceae
Cyanophora
Gloeochaete
Cyanoptyche
Glaucocystis
P
Chl
Cha
LP
CY
SCRP
BA
FL
Phagotrophic Eukaryote
Cyanobacteria
Primary Endosymbiosis
Primary Plastid
Archaeplastida comon ancestor
44/45
48[2/3]
PBC
PBX-Crypto
GSM1
GSM1
MAM-A
MAM-B
GLX
Class-B
KNOX1
KNOX2
GLX
Class-B
?
KNOX1
KNOX2
BELL
KNOX-Red1
KNOX-Red2
PBX-Red
KNOX-Glauco
PBX-Glauco

Fig. S6. Alignment of Class-B TALE proteins in volvocales. Short motifs are conserved among all members in this class over the entire length of the sequence.

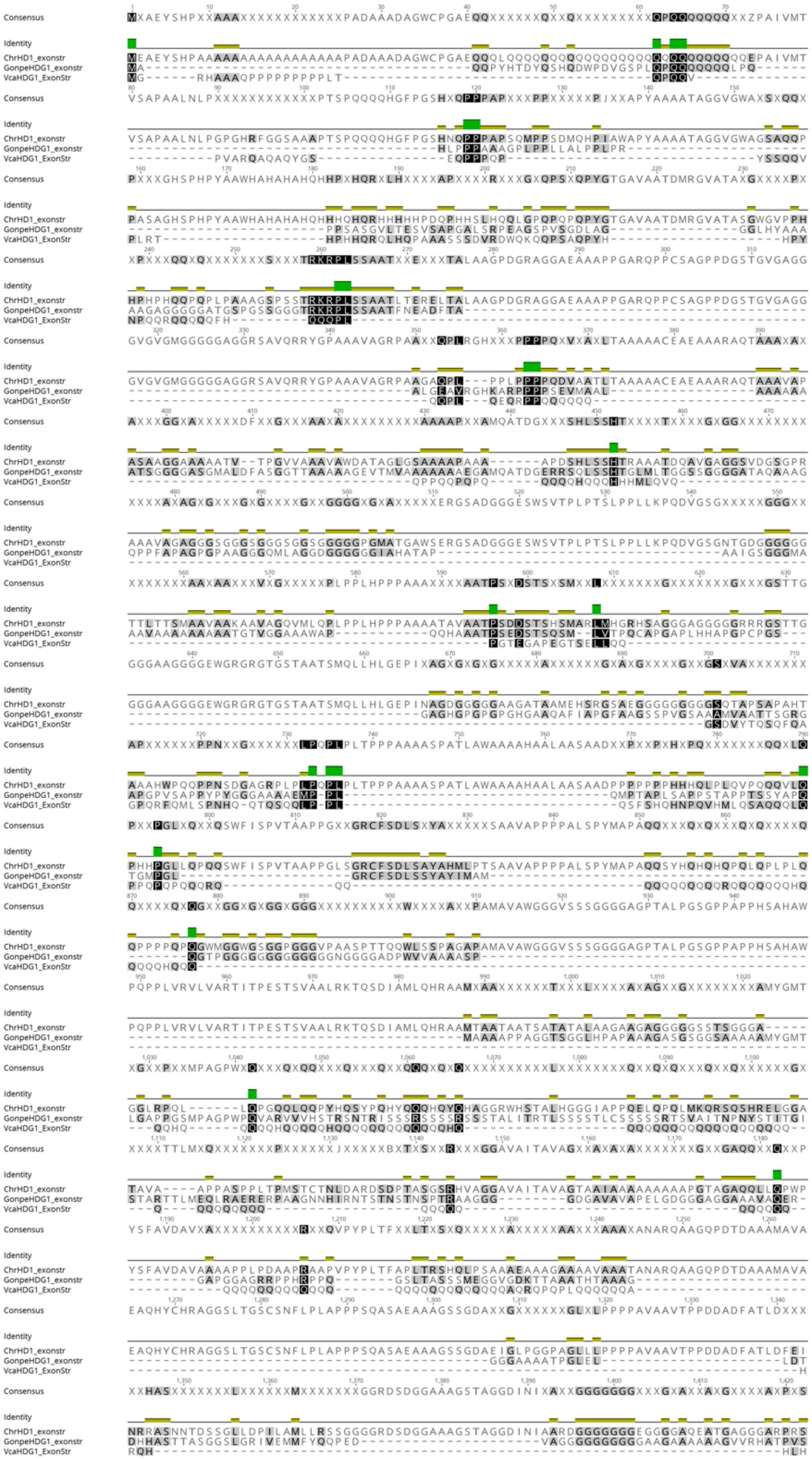

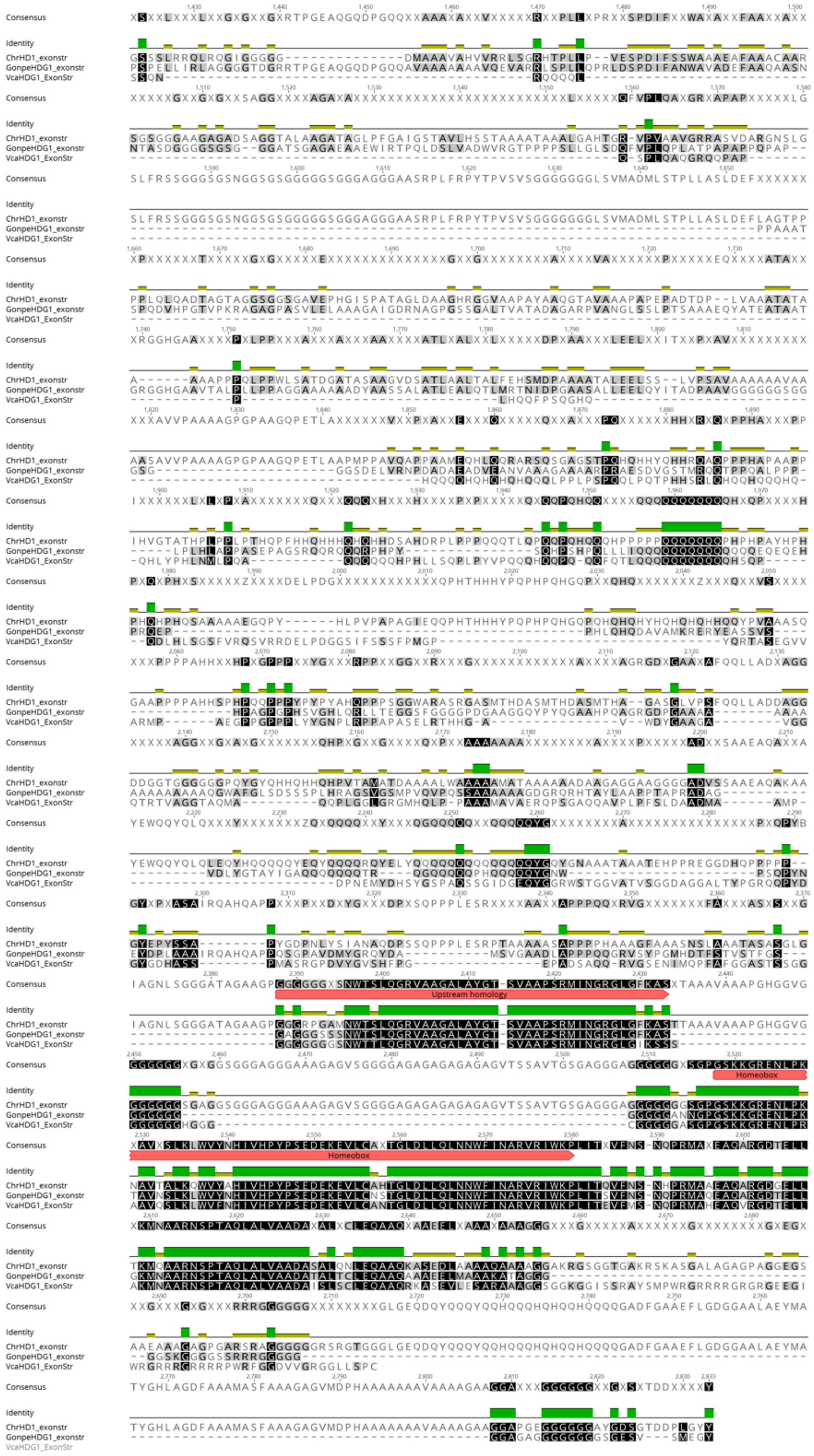
Consensus
Identity
ChrHD1_exonstr
GonpeHDG1_exonstr
VcaHDG1_ExonStr
Upstream homology
Homeobox

Fig. S7. Alignment of Mam-A TALE proteins in mamiellophyceae. Short motifs (Box1-4) are conserved among all members in this class over the entire length of the sequence. Red reverse triangle at 548-549 shows the truncation position of Micco_Mam-A-tr used in Yeast-two-hybrid analysis.

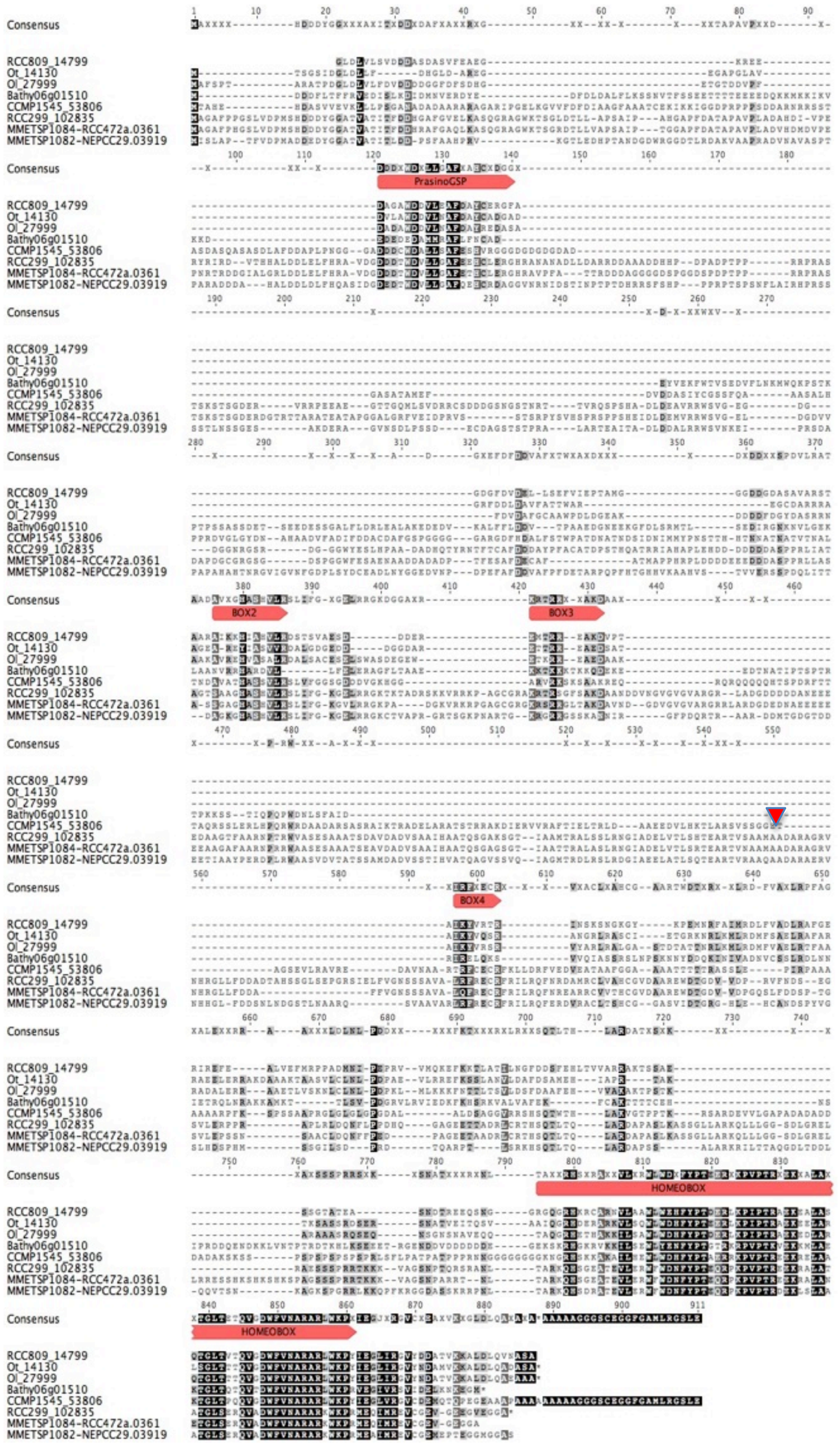

Fig. S8. Alignment of Mam-B TALE proteins in mamiellophyceae. A conserved motif is found between 180-197 amino acids in the alignment. Red reverse triangle at 100-101 shows the truncation position of Ostta_Mam-B-tr used in Yeast-two-hybrid analysis. Homology is restricted to a single homology-A domain ouside the homeodomain.

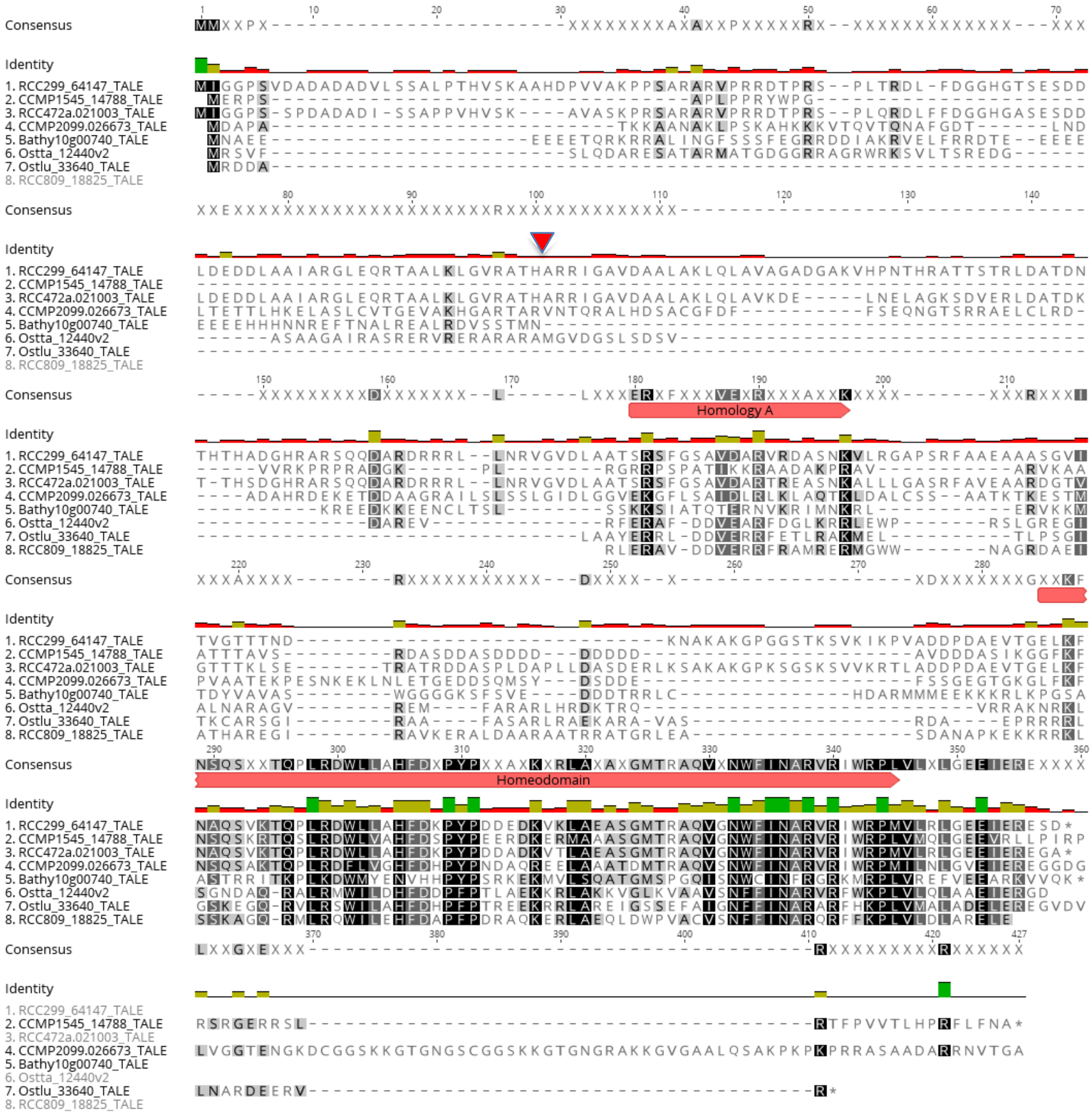

Fig. S9. Full-length proteins are necessary for mamiellophyceae non-KNOX TALE proteins to form heterodimers. Left and Right: Yeast-two-hybrid assays on Ade-/His-/Leu-/Trp- medium. The construct information for the prey conjugated with the GAL4 DNA-binding domain and for the bait conjugated with the GAL4 transcriptional activation domain is given in the table below.

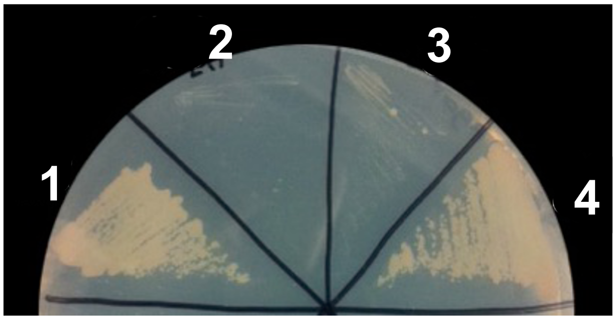

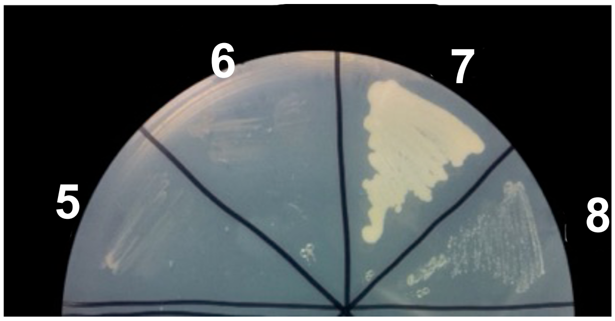

Sector	DBD	AD	Sector	DBD	AD
1	Ostta_KNOX	Ostta_Mam-A	5	Micco_Mam-B	T-Ag
2	Ostta_KNOX	Ostta_Mam-B-tr	6	Micco_Mam-A-tr	Micco_KNOX
3	Ostta_Mam-A	Ostta_Mam-B-tr	7	Micco_Mam-B	Micco_KNOX
4	Ostta_Mam-A	Ostta_KNOX	8	Micco_Mam-B	Micco_Mam-A-tr

Fig. S10. TALE interaction network defined by this study using yeast-two-hybrid assays. (A) Summary diagram for the TALE interaction network. (B) Yeast-two-hybrid assays for the cross-species interaction of TALE proteins. Only one of the possible reciprocal combinations of GAL4 domain conjugations is provided for simplicity. Large X indicates no yeast in the sector. -LTHA: Leu-/Trp-/His-/Ade- medium; -LT: Leu-/Trp- medium.

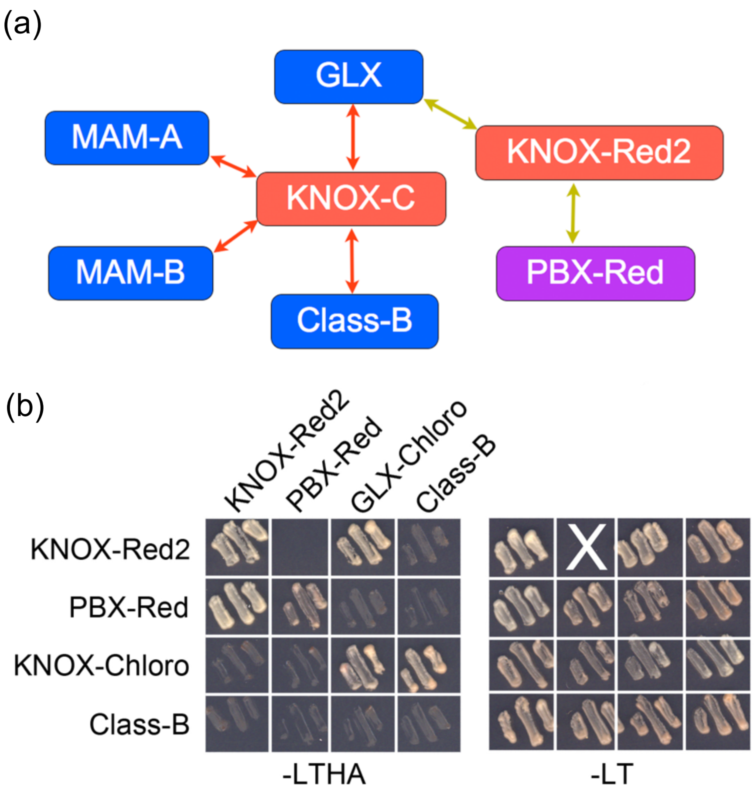

Fig. S11. Identification of MEIS homologs in choanoflagellates.

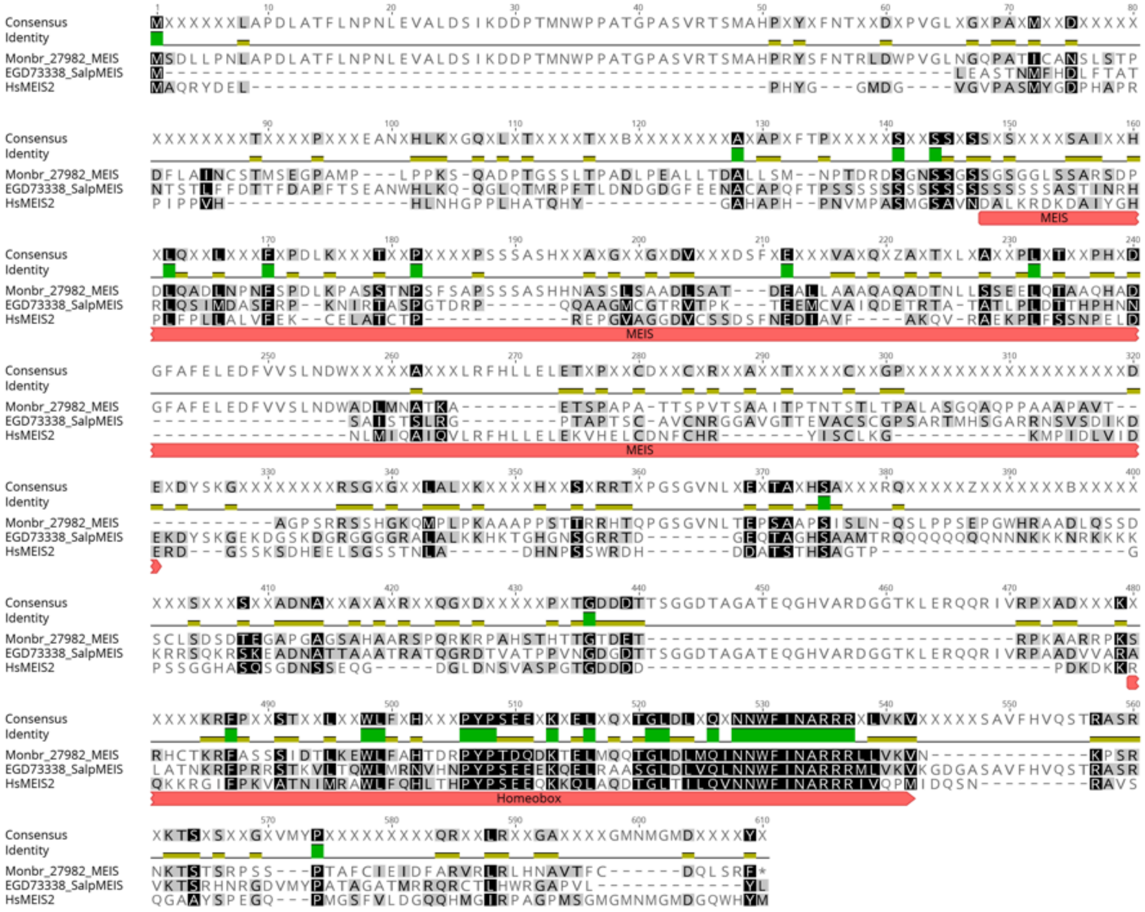

Fig. S12. Identification of PBX homologs in choanoflagellates.

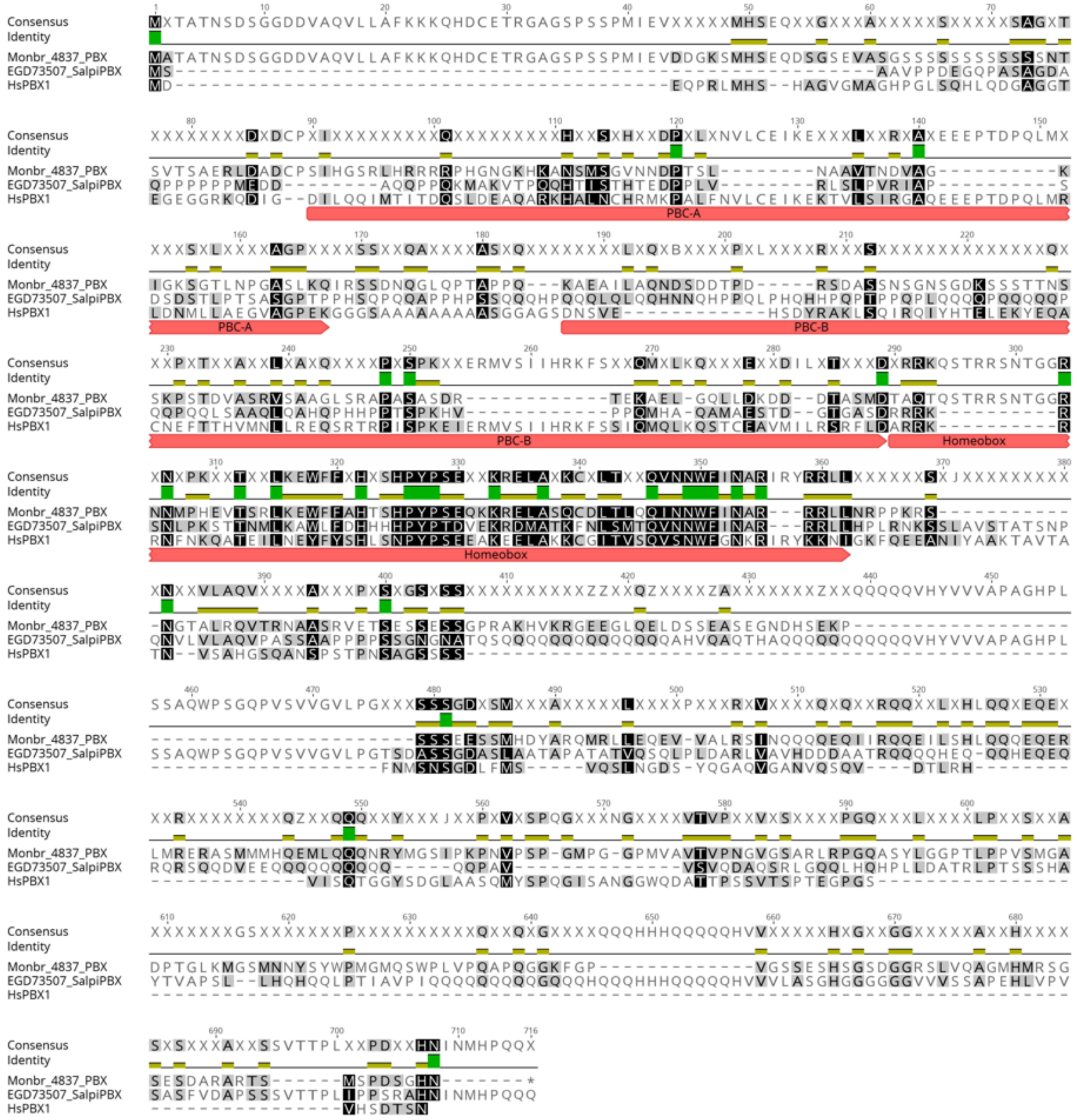

Fig. S13. Alignment of the MEIS homologs in Amorphea.

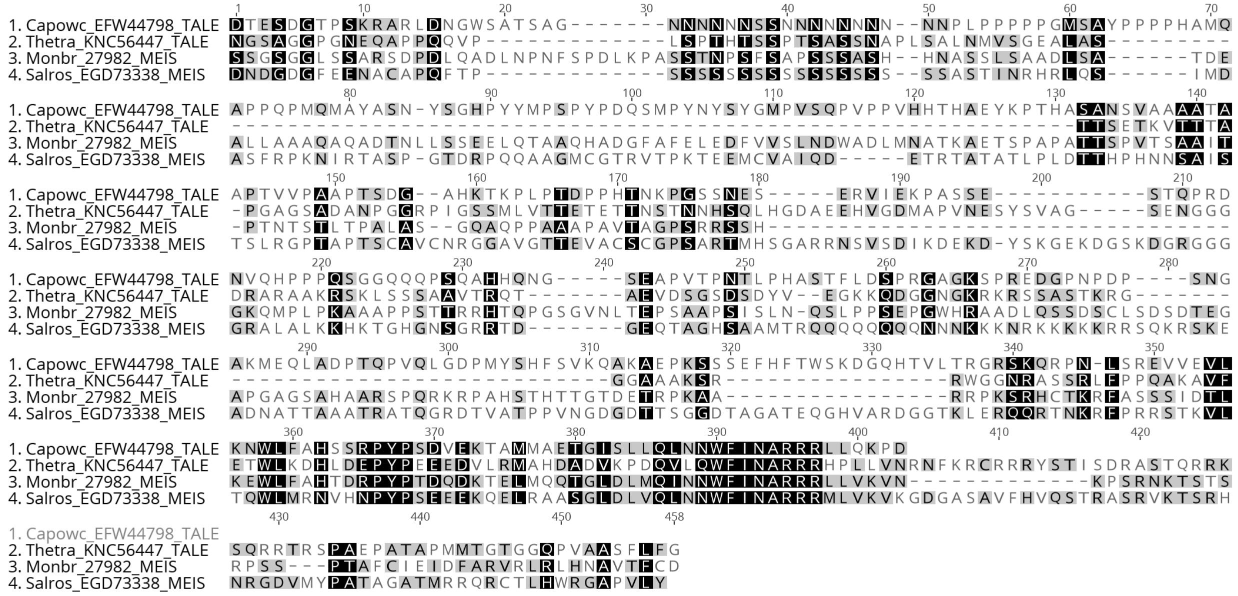

Fig. S14. Alignment of the PBX homologs in Amorphea.

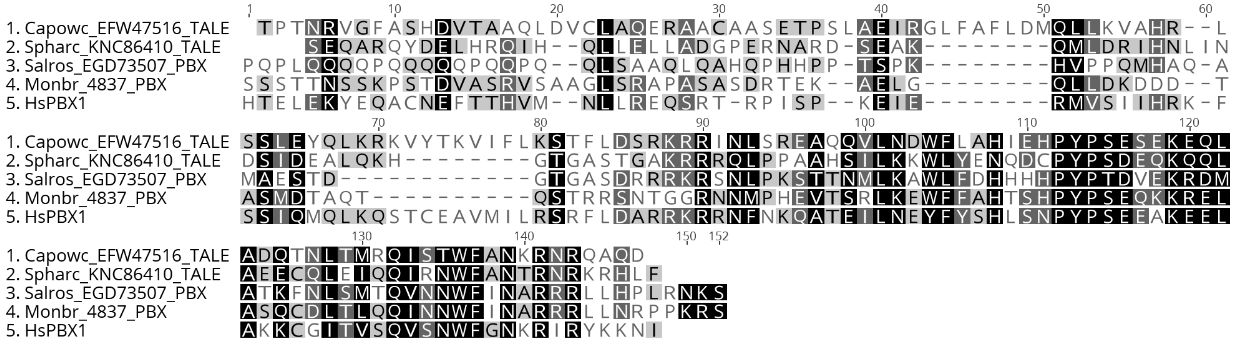

Fig. S15. ELK-domain alignment.

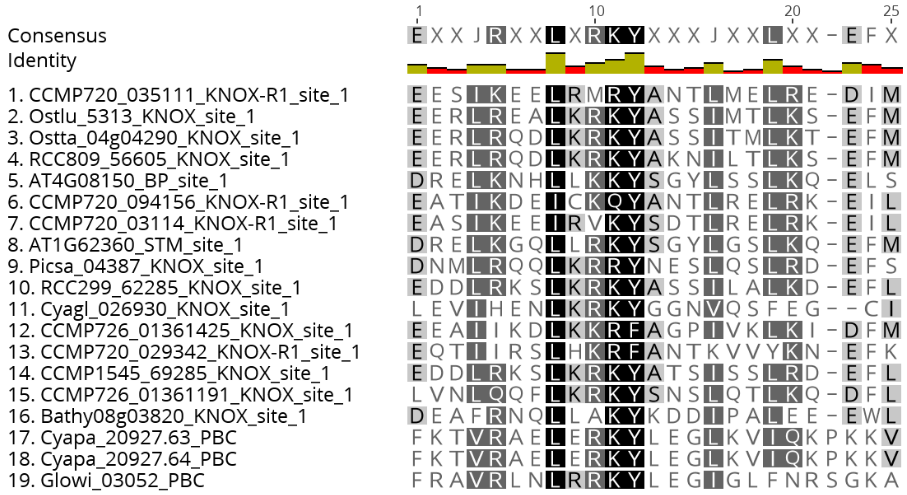

Supplement: Supplementary file 3 — Figure S1. Alignment of TALE homeodomain sequences of the Archae-algal collection. Figure S2. Homology domain alignment of KNOX homologs. Figure S3. GLX class is defined by PBL-Chloro domain. Figure S4. Extensive homology of Picsa_04995 and Klefl_00021_0250 to Chocr_41034 indicates their classification as PBX-Red. Figure S5. Intron-retention pattern suggests parallel evolution of KNOX and non-KNOX group classes from common duplicated TALE ancestors. Figure S6. Alignment of Class-B TALE proteins in volvocales. Figure S7. Alignment of Mam-A TALE proteins in mamiellophyceae. Figure S8. Alignment of Mam-B TALE proteins in mamiellophyceae. Figure S9. Full-length proteins are necessary for mamiellophyceae non-KNOX TALE proteins to form heterodimers. Figure S10. TALE interaction network defined by this study using yeast-two-hybrid assays. Figure S11. Identification of MEIS homologs in choanoflagellates. Figure S12. Identification of PBX homologs in choanoflagellates. Figure S13. Alignment of the MEIS homologs in Amorphea. Figure S14. Alignment of the PBX homologs in Amorphea. Figure S15. ELK-domain alignment. (PDF 10098 kb) [file 12915_2018_605_MOESM3_ESM.pdf]
