# Supplementary material for: Common ancestry of heterodimerizing TALE homeobox transcription factors across Metazoa and Archaeplastida
Source: BMC Biol. 2018 Nov 5;16:136. doi: 10.1186/s12915-018-0605-5 (PMC6219170; doi:10.1186/s12915-018-0605-5)
Supplement: Supplementary file 4 — Data S1. Results of HMM-motif search for KN-A, KN-B, and PBL-Red domains. Data S2. Alignment of the KN-A, KN-B, and PBL-Red domains. (PDF 66 kb) [file 12915_2018_605_MOESM4_ESM.pdf]

Additional file 4 for Joo et al.

Article title: Evolution of heterodimerizing TALE homeobox transcription factors as a developmental mechanism for the haploid-to-diploid transition.

Authors: Sunjoo Joo, Ming Hsiu Wang, Gary Lui, Jenny Lee, Andrew Barnas, Eunsoo Kim, Sebastian Sudek, Alexandra Z. Worden, and Jae-Hyeok Lee.

Supplemental Data S1-S2

Data S1. HMM-motif search result

```
Query:       KNA_JW2  [M=31]
Scores for complete sequences (score includes all domains):
   --- full sequence ---   --- best 1 domain ---    -#dom-
    E-value  score  bias    E-value  score  bias    exp  N  Sequence
    ------- ------ -----    ------- ------ -----   ---- --  -------------------
    2.1e-12   41.2   0.0    8.6e-12   39.4   0.0    2.1  1  RCC809_56605_KNOX
    2.3e-12   41.2   0.2    1.1e-11   39.0   0.0    2.3  1  Ostlu_5313_KNOX
    3.5e-12   40.6   0.5    2.6e-11   37.9   0.1    2.5  2  Ostta_04g04290_KNOX
    2.7e-10   34.7   1.3    5.5e-10   33.7   0.0    2.2  1  Picsa_04387_KNOX
    8.3e-10   33.1   0.8      2e-09   31.9   0.0    2.1  1  Aceac_AAD51632_KNOX1
    8.7e-10   33.1   1.1    2.9e-09   31.4   0.2    2.4  1  AT1G62360_STM
    1.8e-09   32.1   1.1    5.4e-09   30.6   0.1    2.5  1  RCC299_62285_KNOX
    1.9e-09   32.0   0.2    7.3e-09   30.2   0.0    2.1  1  AT4G08150_BP
    2.8e-09   31.5   0.0    5.3e-08   27.5   0.0    2.9  2  Pp3c21_10320_MKN5
    6.1e-09   30.4   0.0    2.3e-08   28.6   0.0    2.0  1  Pp3c18_18720_MKN2
    1.3e-08   29.4   0.0    3.9e-08   27.9   0.0    1.8  1  Pp3c6_1550_MKN6v2
    1.5e-08   29.2   0.3      6e-08   27.3   0.3    2.1  1  CCMP1545_69285_KNOX
    1.5e-08   29.2   0.0    4.6e-08   27.7   0.0    1.9  1  Pp3c5_26440_MKN1
    1.9e-08   28.9   0.3    8.3e-08   26.9   0.3    2.2  1  Cre08.g375400_KNOX/GSM1
    2.1e-08   28.8   0.8    9.1e-08   26.7   0.0    2.4  2  AT5G25220_KNAT3
    2.3e-08   28.6   0.5    1.7e-07   25.9   0.0    2.7  1  Cymte_Sc9672_1_KNOX
    1.2e-07   26.4   0.2    6.6e-07   24.1   0.1    2.3  1  Cz15g18055v2_KNOX
      3e-07   25.1   0.9    8.7e-05   17.4   0.0    3.8  3  Klefl00118_0070_KNOX2
      5e-07   24.5   0.0    1.8e-06   22.7   0.0    2.0  1  Cymte_Sc2807_10_KNOX
    1.6e-06   22.9   0.0      5e-06   21.3   0.0    1.9  1  CCMP726_01361191_KNOX
    2.6e-06   22.2   0.1    1.3e-05   20.0   0.1    2.3  1  Klefl00113_0180_KNOX1
      4e-06   21.6   0.0    1.7e-05   19.6   0.0    2.0  1  Cyagl_026930_TALE
    4.7e-06   21.4   0.0    1.2e-05   20.1   0.0    1.7  1  CCMP720_03114_KNOX
    8.6e-06   20.6   0.0    2.9e-05   18.9   0.0    2.0  1  Bathy08g03820_KNOX
      1e-05   20.3   0.1    0.00014   16.8   0.0    2.5  2  Dunsa_0012s00032v2_KNOX
    1.7e-05   19.6   2.0    1.9e-05   19.5   0.1    2.1  1  Pycno_01672_KNOX
    3.1e-05   18.8   0.0    8.9e-05   17.4   0.0    1.8  1  CCMP720_035111_KNOX
    4.5e-05   18.3   0.2    0.00014   16.8   0.2    1.9  1  Gonpe_2g991_KNOX
    0.00024   16.1   0.8     0.0014   13.7   0.0    2.8  1  Cyapa_21179.70_KNOX
    0.00047   15.1   0.0     0.0015   13.6   0.0    2.0  1  Klefl00157_0150_TALE2
     0.0019   13.2   0.0     0.0059   11.7   0.0    1.9  1  CCMP726_01351541_KNOX
     0.0019   13.2   0.0     0.0059   11.7   0.0    1.9  1  CCMP726_01357749_KNOX
```

```
Query:       KNB_JW2  [M=22]
Scores for complete sequences (score includes all domains):
   --- full sequence ---   --- best 1 domain ---    -#dom-
    E-value  score  bias    E-value  score  bias    exp  N  Sequence
    ------- ------ -----    ------- ------ -----   ---- --  ----------------
    3.1e-08   27.5   0.9    3.9e-07   24.2   0.2    3.2  2  Aceac_AAD51632_KNOX1
    2.5e-07   24.8   0.7    2.1e-06   22.0   0.4    2.7  1  AT4G08150_BP
    4.2e-07   24.1   0.6      2e-06   22.1   0.2    2.4  1  AT1G62360_STM
    8.1e-07   23.3   0.2    3.4e-06   21.4   0.2    2.2  1  Pp3c5_26440_MKN1
    2.1e-06   22.0   0.2    1.1e-05   19.8   0.1    2.3  1  CCMP726_01361191_KNOX
    1.3e-05   19.6   3.4      2e-05   19.1   0.2    3.0  2  Ostlu_5313_KNOX
    2.3e-05   18.8   0.0    0.00012   16.7   0.0    2.2  1  CCMP720_029342_KNOX
    2.6e-05   18.7   0.0    0.00024   15.8   0.0    2.6  2  Pp3c18_18720_MKN2
    3.5e-05   18.3   0.2    0.00015   16.4   0.2    2.1  1  Ostta_04g04290_KNOX
    8.6e-05   17.1   0.2    0.00044   15.0   0.2    2.3  1  AT5G25220_KNAT3
    9.2e-05   17.0   0.5    0.00043   15.0   0.5    2.3  1  Cz15g18055v2_KNOX
     0.0001   16.9   0.1     0.0012   13.6   0.0    2.7  2  Picsa_0325_TALE-R1
    0.00024   15.8   0.3     0.0014   13.4   0.2    2.5  1  RCC809_56605_KNOX
    0.00027   15.6   2.6    0.00042   15.0   0.2    2.6  2  Galsu_EME30635_TALE
    0.00035   15.3   0.0     0.0014   13.4   0.0    2.2  1  Pp3c6_1550_MKN6v2
    0.00038   15.2   0.0     0.0014   13.5   0.0    2.0  1  Cyapa_21179.70_KNOX
     0.0004   15.1   0.2       0.01   10.8   0.0    3.0  2  Porpu_2271.2_TALE
    0.00043   15.0   0.1     0.0014   13.4   0.1    2.0  1  Pp3c21_10320_MKN5
    0.00046   14.9   0.1     0.0027   12.6   0.1    2.3  1  Pp3c19_5620V_MKN4
    0.00046   14.9   0.8     0.0026   12.6   0.1    2.8  1  RCC299_62285_KNOX
    0.00084   14.1   0.0     0.0047   11.9   0.0    2.4  1  Bathy08g03820_KNOX
    0.00086   14.1   0.6     0.0044   11.9   0.0    2.5  1  Klefl00118_0070_KNOX2
     0.0014   13.5   0.1     0.0087   11.1   0.1    2.5  1  CCMP726_01351541_KNOX
     0.0014   13.4   1.9     0.0035   12.3   0.2    2.5  1  Porum_0247s0008_RKNOX1
     0.0014   13.4   0.5      0.023    9.8   0.1    2.9  2  Picsa_04387_KNOX
     0.0014   13.4   0.1     0.0086   11.1   0.1    2.5  1  CCMP726_01357749_KNOX
     0.0015   13.4   2.2     0.0044   11.9   0.2    2.7  2  Pyrye_g7505_TALE
     0.0017   13.2   0.1      0.011   10.7   0.1    2.4  1  CCMP1545_69285_KNOX
  ------ inclusion threshold ------
      0.021    9.9   3.5      0.016   10.2   0.1    2.7  1  Gonpe_2g991_KNOX


Query:       PBC_JW  [M=67]
Scores for complete sequences (score includes all domains):
   --- full sequence ---   --- best 1 domain ---    -#dom-
    E-value  score  bias    E-value  score  bias    exp  N  Sequence
    ------- ------ -----    ------- ------ -----   ---- --  -------------------
    2.7e-24   79.4   0.3    5.6e-24   78.4   0.3    1.6  1  Cyame_CMR176C_TALE
    1.2e-19   64.5   0.4    3.3e-18   60.0   0.3    2.3  2  Galsu_EME32651v2_TALE
    1.9e-17   57.5   0.3    4.8e-17   56.2   0.3    1.7  1  Chocr_41034_TALE
      3e-17   56.9   1.0      6e-17   55.9   1.0    1.5  1  Picsa_04995_TALE-R2
    3.4e-17   56.7   1.7    7.9e-17   55.5   1.7    1.6  1  Porpu_4416.19_TALE
    4.2e-17   56.4   1.1    6.6e-17   55.8   1.1    1.3  1  Glowi_03052_TALE
    5.6e-17   56.0   0.8    5.6e-17   56.0   0.8    1.8  2  HsPBX1
    6.2e-17   55.9   4.8    6.2e-17   55.9   4.8    1.8  1  Porpu_3495.4_TALE
      9e-17   55.3   3.6    1.8e-16   54.4   3.6    1.5  1  Porpu_2282.2_TALE
    1.1e-16   55.1   6.8    2.2e-16   54.1   6.8    1.5  1  Cyapa_20927.63_PBC1
    2.6e-16   53.8   2.6    5.4e-16   52.9   2.6    1.6  1  Porpu_3495.3_TALE
```

7.4e-16	52.4	0.7	1.5e-15	51.4	0.7	1.5	1	Porpu_677.2_TALE?
5e-15	49.7	0.4	5e-15	49.7	0.4	1.9	2	Accas_4342337_PBX
7.7e-15	49.2	0.6	4.4e-14	46.7	1.1	2.1	2	Porpu_2095.1_TALE?
1.1e-14	48.6	1.0	2.6e-14	47.5	1.0	1.6	1	Caltu_18607_PBX
1.3e-14	48.4	1.0	3.5e-14	47.0	1.0	1.8	1	Caltu_g18607v2_PBX
2.1e-13	44.5	0.1	5.4e-13	43.2	0.1	1.7	1	Klefl00021_0250_TALE1
2.5e-06	21.9	1.6	5.4e-06	20.8	1.6	1.6	1	Cyapa_20927.64_PBC2
4.4e-06	21.1	0.3	7.3e-06	20.4	0.3	1.3	1	Porum_1010s0003_RPBX
2.5e-05	18.7	0.2	5.3e-05	17.6	0.2	1.5	1	Pyrye_con7264

Data S2. Domain alignment in fasta format.

KN-A domain

```
>Calliarthron_tuberculosum_g2262_extraction
MLPHPALALVIRFAFDA--RDADQVELREGD------GTAEALR
>Cyanidizyon_merolae_CMH049C_extraction
VRSHSFFGKLASGVQDV-------VVAPPAD------SSAEELQ
>Galdieria_sulfuraria_EME32194_extraction
IQQHESFVYSFLKALEA------WKQIGWNT-------NHAVIQ
>Picocystis_salinarum_0325_extraction
LTSHPLFPSLLKQF---------RKNAE----------LLEELD
>Klebsormidium_flaccidum_00157_0150_extraction
ASKRRESKERDKQQVECGHNQQEASKGAKKKRERKGGGRQAVSS
>Micromonas_commoda_62285_extraction
VRANPRYPKLLDAYFAC------RRVGADAT-------SKASLA
>Micromonas_pusilla_69285_extraction
MRVHTMYPALLRAYFAC------RSAGADRA-------TELALR
>Ostreococcus_tauri_04g04290_extraction
IRAHPMYARLVEAYYEC------RKIGAHGD-------AAVALE
>Ostreococcus_lucimarinus_g5313_extraction
VRAHPMYPRLVEAYYEC------RQIGAEGD-------VLEALD
>Ostreococcus_sp_RCC809_56605_extraction
IRAHPMYSRLVEAYYEC------RKIGADSE-------TADALE
>Bathycoccus_prasinos_08g03820_extraction
VRTHEKFREYVEIMMTC------RKLTAQTK------RERESLE
>Gonium_pectorale_KXZ56109_extraction
LLHHPRLRELVDAVVGC------RKVVVRSR------EAADELD
>Chlamydomonas_reinhardtii_GSM1_extraction
VQQHEAYPAVVRGTLDV------RKACLPRFPLVAREQAEAELQ
>Volvox_carteri_0053s0055_extraction
FLWHPQKADALLAVFEA------KMAVTDPVN------AATNFT
>Chromochloris_zofingiensis_15g18055v2_extraction
VACHPLYPKIMDAICSC------MKVAQPVE-------VTQQLD
>Pyramimonas_parkeae_01351541_extraction
IGSHPEFKRVVQLYGNI------MRLDGHIT------SMPPDME
>Pyramimonas_parkeae_01361191_extraction
IQKHRLYSRMDTAYYLLGKHDNAMRFGENAD----GDFPLATTQ
>Cymbomonas_tetramitiformis_Sc2807_10_extraction
IITHPLYQRLVKANFER------LKLGQAEE-------AKAGID
>Cymbomonas_tetramitiformis_Sc9672_1v2_extraction
```

```
VMRHPLFPRLVQANFDS------LRIGQLPS-------QVETLN
>Pycnococcus_sp_01672_extraction
VAYHALYLRLVEAHFNV------RRLSEKDS-------SDSVLE
>Picocystis_salinarum_04387_extraction
ICAHPLYPKLLTAHFEC------KMVGLNEE-------EKASLQ
>Acetabularia_acetabulum_AAD51632_extraction
VIMHPLYPDLVKAIMDC------RKVGGMDE-------SRHHIQ
>Pyramimonas_amylifera_03114_extraction
AYTLQLFCRIVHAYSRSQQNDDSRQMGLLAD-------SEDLLE
>Pyramimonas_amylifera_035111_extraction
AHTLQLFCQNIHKYMRCLQVDGRLLWSSFSS-------SDRLFE
```

```
>Arabidopsis_thaliana_STM_extraction
IMAHPHYHRLLAAYVNC------QKVGAPPE-------VVARLE
>Arabidopsis_thaliana_BP_extraction
IIAHPHYSTLLQAYLDC------QKIGAPPD-------VVDRIT
>Physcomitrella_patens_MKN5_extraction
IIDHPFYPEMVLAHVRV------FKIGAPRR-------LINKLD
>Physcomitrella_patens_MKN2_extraction
IIDHPFYPEMVLAHVRV------FKIGAPRR-------LRRKLD
>Klebsormidium_flaccidum_00113_0180_extraction
IRRHDRYPELVGRLMDC------LKVGASEN-------EIRRLD
>Arabidopsis_thaliana_KNAT3_extraction
ILSHPLYEQLLSAHVAC------LRIATPVD-------QLPRID
>Physcomitrella_patens_MKN1_extraction
IVAHPLYPDLLNAHASC------LRVGTPVD-------QLPHIE
>Physcomitrella_patens_MKN6v2_extraction
IVNHPLYPEMLMNHAAC------LRVGTPVD-------QLPSIE
>Klebsormidium_flaccidum_00118_0070_extraction
IECNQSYPKLIAAHVAC------VQMDTSSP-------RLYQLE
>Cyanoptyche_gloeocystis_026930_extraction
IRKHPLWPSVMTMFRDA------EKSGIKKI-------QLSELP
>Cyanophora_paradoxa_21179.70_extraction
VRSHPSFPLIAEGVRAV------ELAGLLSR-----GHPAAAFP
```

____

KN-B domain

```
>Calliarthron_tuberculosum_g14488_extraction
NSDLLDVFFAALS----AALDQLRQTN
>Calliarthron_tuberculosum_g2262_extraction
QSSSLDIFLSALC----SALREANSAS
>Cyanidizyon_merolae_CMH049C_extraction
ILTILDTLAAAVCYV--RNLQAWVALQ
>Picocystis_salinarum_0325_extraction
DFPELDEFCINYI----ARLKAQREAV
>Klebsormidium_flaccidum_00157_0150_extraction
LQSEGPSFLPPDFL---TDLDGLDANL
>Pyropia_yezoensis_g7505_extraction
D-AAKMEFVSAYM----ALMDVYEARC
>Chondrus_crispus_c1808_extraction
S-VERARFFDTYE----RLIKEYEARC
>Porphyra_umbilicalis_0247s0008_extraction
D-ASKMAFVSSYM----QLMDVYEARC
>Cyanidizyon_merolae_CMR153C_extraction
E-PSRNGFDGRST----GRLGTAETTP
>Galdieria_sulfuraria_EME30635_extraction
N-EDFQHFLETYV----ELLEKYEQRC
>Porphyridium_purpureum_3408.5_extraction
A-LAFRMQSAEYA----ELLGMYESRL
>Porphyridium_purpureum_2271.2_extraction
P-GSSDEWFHTYI----SLIESYKNRL
```

>Porphyridium_purpureum_4494.3_extraction
G-LGLKLSSGVAP----QTMDVFQQRL
>Micromonas_commoda_62285_extraction
G-AELDEFMDNVT----DELTAYAEEL
>Micromonas_pusilla_69285_extraction
G-EEMDDFIARCT----AELRAYSVEL
>Ostreococcus_tauri_04g04290_extraction
S-CDLDEFMRDCT----HELETYVKEL
>Ostreococcus_lucimarinus_g5313_extraction
Q-RDLDRFMRECT----HELESYVKEL
>Ostreococcus_sp_RCC809_56605_extraction
A-LDLDDYMRECT----HELESYVKEL
>Bathycoccus_prasinos_08g03820_extraction
K-QELNAFMEHSC----ENARKFQKEL
>Gonium_pectorale_KXZ56109_extraction
D-PALDLAVNQYI----DLCEAYQAEL
>Chlamydomonas_reinhardtii_GSM1_extraction
N-PGLDEAMGAYV----RVAALYGDEA
>Volvox_carteri_0053s0055_extraction
K-IDPMSFMERAV----GRLNMAHNLH
>Chromochloris_zofingiensis_15g18055v2_extraction
D-EDLDAFMQRYL----QVLASYLREL
>Tetraselmis_astigmatica_021027_extraction
EMEGCRHFVSKAA----EALD---QTL
>Pyramimonas_parkeae_01351541_extraction
C-PELDEFMVLFR----QALERHWVDR
>Pyramimonas_parkeae_01361191_extraction
P-SELDGFMEDCC----KLLETWNDEL
>Cymbomonas_tetramitiformis_Sc2807_10_extraction
D-EELDAFMLEYC----STVETQCGEM
>Cymbomonas_tetramitiformis_Sc9672_1v2_extraction
D-DDLDDFMLNYC----SILESYHEET
>Pycnococcus_sp_01672_extraction
D-HFLNEFMFKYV----MIVDSLHLEL
>Picocystis_salinarum_04387_extraction
E-QEIRKFIQDYC----CTLESLRSDL
>Acetabularia_acetabulum_AAD51632_extraction
D-PELDQFLRQYI----QVLDELHAEL
>Pyramimonas_amylifera_029342_extraction
N-PELQRWRYQDIFGTWKEMESCDRNF
>Pyramimonas_amylifera_03114_extraction
--RNIHGFLASDI-----ALLEVDSSI
>Pyramimonas_amylifera_035111_extraction
E-GEVSDILEKFS----DTWREIESNT
>Pyramimonas_amylifera_094156_extraction
E-KEVSDTCKSMI----STLEDFDFNT
>Arabidopsis_thaliana_STM_extraction
D-PGLDQFMEAYC----EMLVKYEQEL
>Arabidopsis_thaliana_BP_extraction
D-PELDQFMEAYC----DMLVKYREEL

>Physcomitrella_patens_MKN5_extraction
D-PALDHFMRSYV----DMLTKFAEDL
>Physcomitrella_patens_MKN2_extraction
D-PELDHFMRSYV----GVLTKFAEDL
>Klebsormidium_flaccidum_00113_0180_extraction
E-PGLDKVMLIEK----QQLVALKQQL
>Arabidopsis_thaliana_KNAT3_extraction
D-KELDQFMTHYV----LLLCSFKEQL
>Physcomitrella_patens_MKN1_extraction
EKTELDQFMAQYI----MLLCSFKDHL
>Physcomitrella_patens_MKN6v2_extraction
EKVELDRFMTEYT----ALLGDFKDVL
>Klebsormidium_flaccidum_00118_0070_extraction
A-PEVDAFMAECL----AQLGRLKAAL
>Cyanoptyche_gloeocystis_026930_extraction
P-PSVQEFLKKMA----QLLQMQIAAS
>Cyanophora_paradoxa_21179.70_extraction
G-SQLDALVSQYIAALQQVLRSFHGTL
>Gloeochaete_wittrockiana_014496_extraction
P-PETNAGAYNSL---QRALRVQQQTS

____
PBL-Red domain

>HsPBX1_extraction
KLSQI-RQIYHTELEKYEQACNEFTTH--------VMNLLREQSRTR-PIS----------
PKEIERMVSIIHRKFS--------------SIQMQLKQS
>Accas_4342337_extraction
KVKKL-QEAYEVELGQLNLVADQYASH--------VMSLLKAQAATR-PIT----------
DKEQSMKLAVLQERFD--------------YLRTQLRQS
>Guith_137502_extraction
QIKLI-ENQFNSEKCKVDQVCEDVCQR--------MRTILNSQRKTR-HVS----------
EQEENVHCQAIQAKFQ--------------YQEYVNDCL
>Naegr_78561_extraction
RFKEL-QKQYRVECSYIDDALKKWQET--------FERVLNNQSSFR-VVS----------
FEEKHRCKKSLEARFG--------------LMKQLLKDK
>Cyapa_20927.63_extraction
KRAAL-ESWYNTQHRKIEVAEAEYRSK--------IEQELLAQRAFR-TIT----------
PQEERTRLMKVENAFK--------------TVRAELERK
>Glowi_03052_extraction
QRQQL-RVWYDAQLQKIDTAASDYTRQ--------VRAALEKQASLR-PVT----------
PSEVAARVDKIQGYFR--------------AVRLNLRRK
>Caltu_g18607_extraction
NVYKVLQANLSSRLKEVRDSAIMVEDV--------LERRLHRQLHFR-PVP----------
VEEVDRRMEYIKIKFL--------------RARLVIVEK
>Chocr_41034_extraction
NFVAL-KNSLMESLANVRHMAQDAVAE--------METNLAQQLHFR-IVP----------
PEEVHNRLRYIVSRFS--------------KTRKEILHK
>Porpu_2282.2_extraction
HEAEF-EE-FKKRLQLMRARCVAYSFY--------LTEELELQKDFR-LVP----------

DVEIHERRREIVMTFL--------------RARQKLIRS
>Porpu_3495.3_extraction
DDPRL-QE-LLEKLHALRQRCEIYTSI--------VSHELELQSKFR-LVP----------
ELEAREREGEVVKAFL--------------RARQTLIRL
>Porpu_4416.19_extraction
GDELM-NS-LIERLQMMRQRCELLSAG--------VVEELLLQSKFR-LVT----------
PEEILERRARIVQLFL--------------DARNRLVEN
>Porpu_3495.4_extraction
E-RAVFQQ-IRNVLQRLREVHGKWMKK--------VQHEICAQREFR-PVS----------
KVEVMFWHNRITAICR--------------AERVRIMQL
>Porpu_2095.1_extraction
ENPGTAKASSQTVMQKMEEIRTVFQAA--------IIEELQEQSKCR-IIS----------
PGEEKERLLAVAAIARAH------------AVRGENVRK
>Porpu_677.2_extraction
GHLELSESLDHHVQKKLKEIRLRYHYL--------MEHELRLQAEFR-IVR----------
DAEKHGRLHAITALTADY------------KARGEALEA
>Galsu_EME32651v2_extraction
Q-KVMWKNWLIDLKTRWSDTVHHYMSL--------MLEELKQQRRFR-TIT----------
EEEVQSWIHPMMQTFE--------------NIW-NIIRQ
>Cyame_CMR176C_extraction
RNLALEYDGPYKDLVRLQAHCLANQVEQLWAESP-CRHEARAPVATRHSVSASASLATAS-
AAEPRDTLYRLPTNVVQGACDASASDSNTXAARCKRKRR
>Picsa_04995_extraction
ETSCPWERTANGNWDEDNWEAEVRESR---------FFEATEQLLLE-CLAYQKTC-----
SRQMQEKEPSFPHSRNECDGNQPTDSRCA-VPKDESHQH
>Klefl00021_0250_extraction
KTGRTGGTAALAEETRVGQTVEATRRGW-------MEEEGGTGREKR--
VGETGGTGGTGXPAEERDERERSRGKRSIEESSEEQD-----EARPRKRGG
