# Supplementary material for: Common ancestry of heterodimerizing TALE homeobox transcription factors across Metazoa and Archaeplastida
Source: BMC Biol. 2018 Nov 5;16:136. doi: 10.1186/s12915-018-0605-5 (PMC6219170; doi:10.1186/s12915-018-0605-5)
Supplement: Supplementary file 5 — Method S1. Collecting TALE homeobox protein sequences. Method S2. Phylogenetic reconstruction. Method S3. Homology motif/domain search. Method S4. Intron comparison. Method S5. Cloning of Yeast-two-hybrid constructs. (PDF 57 kb) [file 12915_2018_605_MOESM5_ESM.pdf]

Additional file 5 for Joo et al.

Article title: Evolution of heterodimerizing TALE homeobox transcription factors as a developmental mechanism for the haploid-to-diploid transition.

Authors: Sunjoo Joo, Ming Hsiu Wang, Gary Lui, Jenny Lee, Andrew Barnas, Eunsoo Kim, Sebastian Sudek, Alexandra Z. Worden, and Jae-Hyeok Lee.

Supplemental Methods S1-S4

Method S1. Collecting TALE homeobox protein sequences

Twenty six available algal genomes, and 16 additional transcriptomes from the Marine Microbial Eukaryote Transcriptome Sequencing Project (MMETSP) dataset (Keeling et al., 2014) (Details in Table S1), were searched by Hmmer for the HD/HD_KN (PF00046/PF05920) and by BLASTP/TBLASTN using published algal homeobox sequences to collect TALE homeobox. Homeodomain sequences were extracted from the collection and aligned using the MAFFT algorithm. Most TALE proteins were identified by the three amino-acid insertions between positions 23/24 in the 60 amino acid-long typical homeodomain (Bürglin, 1997). More divergent sequences with extensive gaps in the alignment could not be ascertained for their classes, therefore, we used secondary structure modeling against the 3D structure database at the SWISS-MODEL site (https://swissmodel.expasy.org/interactive) to evaluate whether the typical TALE homeodomain structure is retained and whether the introduced indels preserve or distort the known homeodomain structure (Biasini et al., 2014). After excluding 6 sequences of high similarity in order to prevent oversampling of a particular taxa, the resulting TALE alignment of 96 TALE homeodomains contained one conserved insertion in the TALE loop between sites b/c among the GLX and Mam-A sequences in Chlorophyta, and two singleton insertions elsewhere that were excluded for phylogenetic reconstructions in the final alignment (S1 Fig). The final alignment includes four *Arabidopsis thaliana* (STM, BP, KNAT3, and BEL1) and six *Physcomitrella patens* TALEs as land plant references, three Human (MEIS2, PKNOX1 and PBX1) and five protozoan TALEs from *Guillardia theta*, *Acanthamoeba castellani, and Naegleria gruberi* as references outside Archaeplastida (S2 Spreadsheet).

Method S2. Phylogenetic reconstruction

A phylogenetic reconstruction was performed using Bayesian (MrBayes; Ronquist et al., 2012) and Maximum-likelihood (PhyML, RAxML, and IQ-TREE; Guindon et al., 2010; Rokas, 2011; Minh et al., 2013) methods. IQ-TREE yielded a strongly supported consensus unrooted tree with Ultrafast bootstrap test score with '-bb 2000 -bi 500' option, whereas the other methods produced trees with poorly resolved clade structure. The LG+R5 model was chosen according

to Bayesian Information Criteria (Luo et al., 2010). Shimodaira-Hasegawa-like (SH) and Bayesian approximate likelihood-ratio tests were performed by IQ-TREE with "-alrt 1000" and "abayes" options (Anisimova et al., 2011). A score of 95% in Ultrafast bootstrap and 80% in SH-alrt was considered highly significant recommended by the software authors. Classification relied on phylogenetic identification of clades with high bootstrap support in more than one tree topology test as well as ad hoc homology-motif searches to identify shared domain structure among the collected homeobox proteins.

Method S3. Curation of gene models

Many sequences lacked homology domains outside the homeodomain, possibly due to incomplete sequence information, lineage- or gene-specific divergence, or false phylogenetic association caused by limited sequence information. To locate possible missing or very divergent domains, we used alignment as a tool, generating extensive alignments combining ranging from three to 20 sequences. All the alignments were manually inspected for the identified motif/domains to be correctly aligned. Sequences with large insertions or deletions were compared with the original genome annotations to detect possible alternative splice sites. When a better gene model was identified than that deposited in GenBank, we used it. These sequences are denoted with "v2" in figures and the protein sequences are provided in S2 Spreadsheet. When necessary, we amended the homeodomain alignment and updated the phylogenetic analysis.

Method S4. Cloning of Yeast-two-hybrid constructs

Micco_62153 and Picsa_04684 contained a single intron, whereas all the other nine genes lacked an intron in the entire open reading frame. For cloning of Micco_62153, we synthesized the middle fragment lacking the intron and ligated them via *Xho*I and *Cla*I sites. For cloning of Picsa_4684, 5'- and 3'-side exons were amplified separately and combined using PCR. Using 5'-*EcoR*I or *Mfe*I and 3'-*BamH*I, *Bgl*II or *Xho*I, cloned DNA fragments were placed in-frame to pGBKT7 and pGADT7 vectors at the *EcoR*I/*BamH*I or *EcoR*I/*Sal*I sites (Clonetech).

References in the supporting information

Biasini M, Bienert S, Waterhouse A, Arnold K, Studer G, Schmidt T, Kiefer F, Gallo Cassarino T, Bertoni M, Bordoli L, et al. 2014. SWISS-MODEL: modelling protein tertiary and quaternary structure using evolutionary information. Nucleic Acids Research 42: W252–8.

Blanc G, Agarkova I, Grimwood J, Kuo A, Brueggeman A, Dunigan DD, Gurnon J, Ladunga I, Lindquist E, Lucas S, et al. 2012. The genome of the polar eukaryotic microalga Coccomyxa subellipsoidea reveals traits of cold adaptation (J Sullivan, Ed.). Genome

Biology 13: R39–552.

Blanc G, Duncan G, Agarkova I, Borodovsky M, Gurnon J, Kuo A, Lindquist E, Lucas S, Pangilinan J, Polle J, et al. 2010. The Chlorella variabilis NC64A genome reveals adaptation to photosymbiosis, coevolution with viruses, and cryptic sex. THE PLANT CELL ONLINE 22: 2943–2955.

Bürglin TR. 1997. Analysis of TALE superclass homeobox genes (MEIS, PBC, KNOX, Iroquois, TGIF) reveals a novel domain conserved between plants and animals. Nucleic Acids Research 25: 4173–4180.

Foflonker F, Price DC, Qiu H, Palenik B, Wang S, Bhattacharya D. 2015. Genome of the halotolerant green alga Picochlorum sp. reveals strategies for thriving under fluctuating environmental conditions. (JE Hallsworth, Ed.). Environmental Microbiology 17: 412–426.

Gao C, Wang Y, Shen Y, Yan D, He X, Dai J, Wu Q. 2014. Oil accumulation mechanisms of the oleaginous microalga Chlorella protothecoides revealed through its genome, transcriptomes, and proteomes. BMC genomics 15: 582.

Guillou, Eikrem, Massana, Romari, Vaulot. 2004. Diversity of Picoplanktonic Prasinophytes Assessed by Direct Nuclear SSU rDNA Sequencing of Environmental Samples and Novel Isolates Retrieved from Oceanic and Coastal Marine Ecosystems. Annals of Anatomy 155: 22–22.

Guindon S, Dufayard J-F, Lefort V, Anisimova M, Hordijk W, Gascuel O. 2010. New algorithms and methods to estimate maximum-likelihood phylogenies: assessing the performance of PhyML 3.0. Systematic biology 59: 307–321.

Jackson C, Clayden S, Reyes-Prieto A. 2015. The Glaucophyta: the blue-green plants in a nutshell. Acta Societatis Botanicorum Poloniae 84: 149–165.

Keeling PJ, Burki F, Wilcox HM, Allam B, Allen EE, Amaral-Zettler LA, Armbrust EV, Archibald JM, Bharti AK, Bell CJ, et al. 2014. The Marine Microbial Eukaryote Transcriptome Sequencing Project (MMETSP): illuminating the functional diversity of eukaryotic life in the oceans through transcriptome sequencing. (RG Roberts, Ed.). PLoS Biol 12: e1001889.

Lopes Dos Santos A, Pollina T, Gourvil P, Corre E, Marie D, Garrido JL, Rodríguez F, Noël M-H, Vaulot D, Eikrem W. 2017. Chloropicophyceae, a new class of picophytoplanktonic prasinophytes. Scientific Reports 7: 14019.

Luo A, Qiao H, Zhang Y, Shi W, Ho SY, Xu W, Zhang A, Zhu C. 2010. Performance of criteria for selecting evolutionary models in phylogenetics: a comprehensive study based on simulated datasets. BMC evolutionary biology 10: 242.

Minh BQ, Nguyen MAT, Haeseler von A. 2013. Ultrafast approximation for phylogenetic bootstrap. Molecular Biology and Evolution 30: 1188–1195.

Rokas A. 2011. Phylogenetic analysis of protein sequence data using the Randomized Axelerated Maximum Likelihood (RAXML) Program. (FM Ausubel, R Brent, RE Kingston, DD Moore, JG Seidman, JA Smith, and K Struhl, Eds.). Current protocols in molecular biology Chapter 19: Unit19.11–19.11.14.

Ronquist F, Teslenko M, van der Mark P, Ayres DL, Darling A, Höhna S, Larget B, Liu L, Suchard MA, Huelsenbeck JP. 2012. MrBayes 3.2: efficient Bayesian phylogenetic inference and model choice across a large model space. Systematic biology 61: 539–542.

Wilhelmsson PKI, Mühlich C, Ullrich KK, Rensing SA. 2017. Comprehensive Genome-Wide Classification Reveals That Many Plant-Specific Transcription Factors Evolved in Streptophyte Algae. Genome Biology and Evolution 9: 3384–3397.
